# Health behaviours the month prior to COVID-19 infection and the development of self-reported long COVID and specific long COVID symptoms: a longitudinal analysis of 1581 UK adults

**DOI:** 10.1186/s12889-022-14123-7

**Published:** 2022-09-09

**Authors:** Elise Paul, Daisy Fancourt

**Affiliations:** Department of Behavioural Science and Health, University College London, 1-19 Torrington Place, London, WC1E 7HB UK

**Keywords:** COVID-19, Long COVID, Health behaviours

## Abstract

**Background:**

Demographic and infection-related characteristics have been identified as risk factors for long COVID, but research on the influence of health behaviours (e.g., exercise, smoking) immediately preceding the index infection is lacking. The aim of this study was to examine whether specific health behaviours in the month preceding infection with COVID-19 act as upstream risk factors for long COVID as well as well as three specific long COVID symptoms.

**Methods:**

One thousand five hundred eighty-one UK adults from the UCL COVID-19 Social Study and who had previously been infected with COVID-19 were analysed. Health behaviours in the month before infection were weekly exercise frequency, days of fresh air per week, sleep quality, smoking, consuming more than the number of recommended alcoholic drinks per week (> 14), and the number of mental health care behaviours (e.g., online mental health programme). Logistic regressions controlling for covariates (e.g., COVID-19 infection severity, socio-demographics, and pre-existing health conditions) examined the impact of health behaviours on long COVID and three long COVID symptoms (difficulty with mobility, cognition, and self-care).

**Results:**

In the month before infection with COVID-19, poor quality sleep increased the odds of long COVID (odds ratio [OR]: 3.53; (95% confidence interval [CI]: 2.01 to 6.21), as did average quality sleep (OR: 2.44; 95% CI: 1.44 to 4.12). Having smoked (OR: 8.39; 95% CI: 1.86 to 37.91) increased and meeting recommended weekly physical activity guidelines (3h hours) (OR: 0.05; 95% CI: 0.01 to 0.39) reduced the likelihood of difficulty with self-care (e.g., washing all over or dressing) amongst those with long COVID.

**Conclusions:**

Results point to the importance of sleep quality for long COVID, potentially helping to explain previously demonstrated links between stress and long COVID. Results also suggest that exercise and smoking may be modifiable risk factors for preventing the development of difficulty with self-care.

**Supplementary Information:**

The online version contains supplementary material available at 10.1186/s12889-022-14123-7.

## Background

Long COVID, which includes both ongoing symptomatic COVID-19 (the presence of symptoms from 4 to 12 weeks post-onset), and post-COVID-19 syndrome (the presence of symptoms > 12 weeks post-onset) [1] is rapidly becoming a major public health concern [2]. The most common symptoms are weakness, fatigue, cognitive difficulties (e.g., concentration and remembering), and breathlessness [3–5]. Findings from studies representative of the general adult population suggest that as of July 2022, around 1.8 million people in the UK self-report COVID-19 symptoms lasting at least 4 weeks (long COVID), with nearly three quarters (72%) reporting that their ongoing symptoms significantly impacted their ability to carry out their day to day activities [5]. Additionally, a large portion of patients suffering from long COVID report reduced quality of life [3, 4].

Most research on long COVID has focused on socio-demographic factors, with female sex, increasing age, and living in a more deprived area as risk factors [5–7]. There is also some emerging evidence of the role of pre-infection health factors such as being obese or overweight and asthma in the development of long COVID [6–10], likely via mechanisms involving chronic systemic inflammation [11]. Focusing on modifiable behavioural risk factors for long COVID is logical, given that several health behaviours, including smoking [12] physical inactivity [13], poor sleep [14, 15] and excessive alcohol consumption not only increase the risk of infectious diseases, but can also impede vaccine response [11].

However, such work on health behaviours and long COVID remains in its infancy. Although COVID-19 symptom severity shows somewhat inconsistent associations with long COVID development [4, 16–18], there is some evidence that health behaviours assessed prior to infection increase the likelihood of long COVID. In a large sample of adults who had tested positive for COVID-19, consistent physical inactivity recorded in the 2 years before COVID-19 infection increased the risk of hospitalisation, ICU admission, and death compared to patients who had consistently met physical activity guidelines [19]. A Mendelian randomisation study found that UK adults predisposed to smoking and to smoking more cigarettes per day were more likely to have been hospitalised and to have died from COVID-19 [20]. Current smoking status increased the likelihood of persistent COVID symptoms (12 weeks or more) in a cross-sectional study of randomly selected community based samples of adults in England [7], it remains unclear whether other behavioural factors occurring before infection with COVID-19influence risk of developing long COVID.

This is important to ascertain, as negative changes have been observed in many of these health behaviours during the pandemic [21, 22] If such behavioural factors are found to increase risk for long COVID it could help inform public health programmes designed to reduce the risk of further cases of long COVID. Thus, the aim of this study was to identify whether specific health behaviours in the month preceding infection with COVID-19 act as upstream and potentially modifiable markers of long COVID as well as three long COVID symptoms.

## Methods

### Study design and participants

Data were drawn from the COVID-19 Social Study; a large panel study of the psychological and social experiences of over 75,000 adults (aged 18+) in the UK during the COVID-19 pandemic. The study commenced on 21 March 2020 and involves online data collection from participants for the duration of the COVID-19 pandemic. Data were initially collected weekly (through August 2020), then monthly thereafter. The study is not random and therefore is not representative of the UK population. But it does contain a well-stratified sample that was recruited using three primary approaches outlined in the Supplemental Materials and in the study User Guide (https://osf.io/jm8ra/). The study was approved by the UCL Research Ethics Committee [approval number 12467/005], performed in accordance with the Declaration of Helsinki, and all participants gave informed consent. Participants were not compensated for participation.

**Fig. 1:**
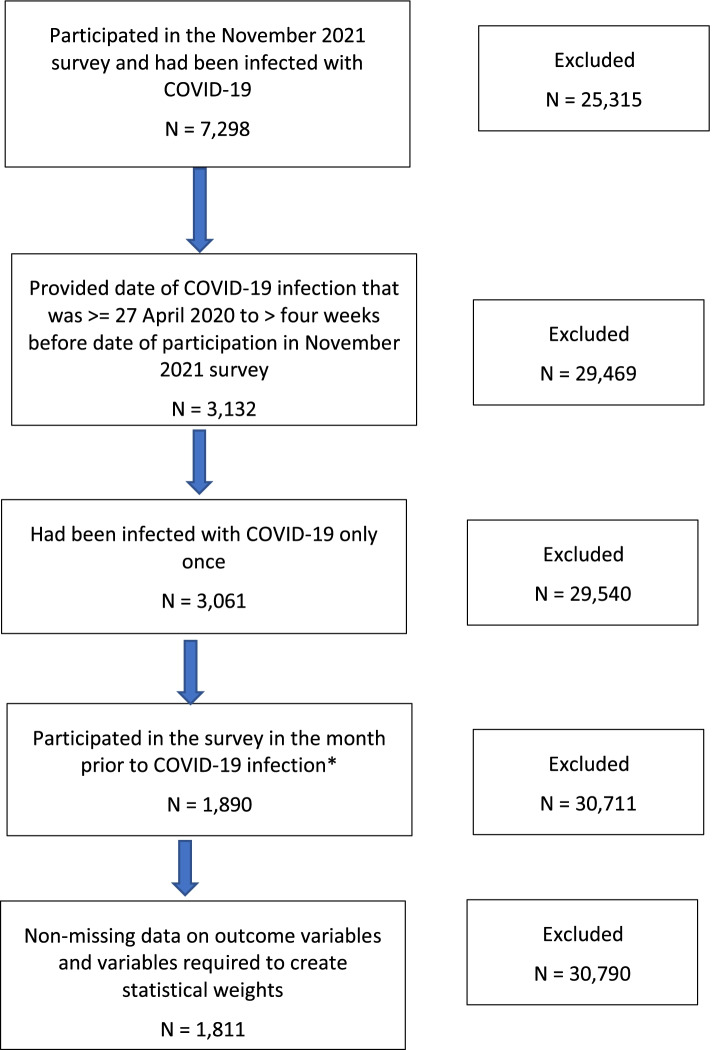
Flow chart of sample selection. * Data from 2 weeks prior to COVID-19 infection were used, and when unavailable, consecutively further weeks prior, up to 6 weeks, were used

We included participants who met the five criteria outlined in Fig. 1. First, participants were included if they had participated in the November 2021 survey and said that they had at some prior point been infected with COVID-19 (see Supplemental Table S1 for question wording). Second, the date given for their COVID-19 infection had to be non-missing and had to be no earlier than 27 April 2020 and at least 5 weeks prior to completion of the specific questions on long COVID. 27 April 2020 was chosen as we were interested in health behaviours in the month prior to COVID-19 infection, and the collection of all individual items comprising these variables commenced 13 April 2020. Five weeks was chosen as the minimum time period as many studies on long COVID apply a threshold of “more than four weeks of symptoms” to be experienced for the term long COVID to be applied [5, 6]. Third, participants who had had COVID-19 only once were included; participants who reported more than one infection were excluded to avoid overlapping symptoms from the two infections. Fourth participants had to have participated in the study in the month prior to the date of their infection to gather health behaviour data. Fifth, participants had to have non-missing data on long COVID outcome variables (presence/absence and specific long COVID symptoms) and study variables required to calculate statistical weights (gender, age, ethnicity, country, and education). The final analytic sample comprised 1581.

We used multiple imputation by chained equations to generate 50 imputed datasets for participants who met all study inclusion criteria but had missing data on other study variables (Supplemental Table S2). Imputation models included all study variables as well as auxiliary variables (e.g., home ownership status, depressive symptoms at baseline). Substantive results using cases without any missing data and the imputed sample were similar (Supplemental Tables S3-S6). See Supplemental Table S7 for a comparison of excluded and included participants on study variables.

### Patient and public involvement

The research questions in the UCL COVID-19 Social Study built on patient and public involvement as part of the UKRI MARCH Mental Health Research Network, which focuses on social, cultural and community engagement and mental health. This highlighted priority research questions and measures for this study. Patients and the public were additionally involved in the recruitment of participants to the study and are actively involved in plans for the dissemination of findings from the study.

### Measures

#### Outcome variables

The presence of long COVID was measured with a binary variable in response to a study-developed question (Supplemental Table S1): no vs yes (formally diagnosed or suspected). Sensitivity analyses tested whether results were consistent when including participants who were “unsure” about whether they had had long COVID within the case group.

To look at the presence of three specific long COVID symptoms, three variables were operationalised from questions assessing the extent to which participants had difficulty with (i) mobility, (ii) cognition, and (iii) self-care (Supplemental Table S7). Response options were treated as binary (present vs absent) in analyses due to low numbers within response categories.

#### Predictor variables

##### Health behaviours

Six health behaviours in the month prior to COVID-19 infection were considered (Supplemental Table S1). Data starting with 2 weeks before the COVID-19 infection were used, and if unavailable, data from 3 weeks, then four, up to 6 weeks (Supplemental Table S8). Weekly exercise frequency was operationalised as none vs < 30 minutes to 2 hours vs 3 hours or more, the latter of which reflects current weekly physical activity recommendations in the UK [23]. A count of the number of days participants had left the house in the past week for at least 15 minutes was also included. Weekly sleep quality was operationalised as very good/good vs average vs not good/very poor. Smoking (non-smoker/no smoking vs any smoking), and a binary variable indicating 14 or more weekly alcoholic drinks (vs <  14) were also included. Fourteen was chosen as the cut-off for alcohol consumption to reflect current recommendations on alcohol intake per week in the UK [24]. Finally, the number of mental health care behaviours was included (e.g., taken medications, spoke to somebody on a support line). Because increasing weight and obesity are associated with long COVID [6, 8, 9], and are also risk factors for chronic disease independent of physical activity [25], we conducted sensitivity analyses with a variable reflecting overweight status collected in June 2020 (slightly underweight or normal weight vs slightly overweight or very overweight).

### Covariates

#### COVID-19 infection variables

COVID-19 infection severity in the first 2 weeks was categorised into (i) asymptomatic, (ii) mild (experienced symptoms but was able to carry on with daily activities), (iii) moderate (experienced symptoms and had to rest in bed), and (iv) severe (participant was hospitalised).

A variable indicating which strain of the virus was dominant in the UK [26] at the time of infection was coded as (0) the original COVID-19 variant (31 January to 31 October 2020, (1) Alpha (1 November 2020 to 30 June 2021), (2) Delta (1 July 2021 to 30 November 2021), and (3) Omicron (1 December 2021 onwards).

### Socio-demographics

Socio-demographics were collected at baseline, which was participants’ first time taking part in the study: gender (male vs female), age (60+, 45–59, 30–44, and 18–29) ethnicity (white vs ethnic minority groups [i.e., Asian/Asian British, etc. See Supplemental Table S1 for a full listing of response options]), education (undergraduate degree or higher, A-levels/vocational training, and up to GCSE (General Certificate of Secondary Education), low income (<£30,0000), employment status (not employed [i.e., at school/ university, unable to work due to disability, etc] vs employed, government’s identified key worker status (vs not a key worker), crowded household (< one room per person), living arrangement (living alone vs living with others but not including children vs living with others, including children), and area of dwelling (urban vs rural).

### Pre-existing health conditions

Participants reported whether they had received a clinical diagnosis of a mental health condition (e.g., depression, anxiety) or chronic physical health condition (e.g., high blood pressure, diabetes). Two binary variables to indicate the presence of pre-existing physical and mental health conditions.

### Statistical analysis

First, binary logistic regression models were fitted to examine associations of health behaviours in the month before infection with COVID-19 and the development of long COVID. Second, binary logistic regression models were fitted to examine associations between health behaviours in the month prior to COVID-19 infection and the presence of each of the three specific long COVID symptoms (difficulty with mobility, cognition, and self-care) amongst participants with long COVID.

For both sets of analyses, Model 1 included only health behaviours in the same model, Model 2 additionally adjusted for COVID-19 infection variables, Model 3 additionally adjusted for socio-demographic characteristics, and Model 4 additionally adjusted for pre-existing health conditions. Robust standard errors were used in all analyses. Coefficients from the binary logistic regressions were exponentiated and presented as odds ratios (OR).

To and increase representativeness of the UK general population, weights were applied throughout all analyses. The sample was weighted to the proportions of gender, age, ethnicity, country, and education in the UK population obtained from the Office for National Statistics [27]. A multivariate reweighting method was implemented using the Stata user written command ‘ebalance’ [28]. Analyses were conducted using Stata version 16 [29].

## Results

One in five (20.48%) in the sample self-reported long COVID (Supplemental Table S9). The most often reported long COVID symptom amongst those with long COVID was difficulty with cognition (62.58%), followed by difficulty with mobility (55.49 (Table 1). People living in crowded accommodation, had a physical or mental health condition, lived with children, had low levels of education or income, and had moderate or severe COVID-19 were all more likely to have developed long COVID.

**Table 1:** Descriptive characteristics and bivariate associations between study variables and long COVID, weighted (*N* = 1581)

	No self-reported long COVID *N* = 1288	Self-reported long COVID *N* = 293	OR	95% CI	
	Prop.	Prop.			
**Long COVID variables**					
Long COVID status					
Yes, but not formally diagnosed	–	81.47%	–	–	
Yes, formally diagnosed	–	18.53%	–	–	
Presence of specific long COVID symptoms			–	–	
Mobility (e.g., walking or climbing steps)	–	55.49%	–	–	
Cognition (e.g., remembering or concentrating)	–	62.58%	–	–	
Self-care (e.g., washing all over or dressing)	–	15.81%	–	–	
**Weekly health behaviours in month prior to COVID-19 infection**					
Exercise frequency (ref no exercise)					
< 30 min to 2 hours	62.53%	66.66%	0.73	0.40	1.35
3h hours	30.07%	22.55%	0.51	0.25	1.08
Days of fresh air (> 15 minutes)	5.72 (1.99)	5.31 (2.35)	0.92	0.83	1.01
Sleep quality (ref very good/good)					
Average	42.80%	45.14%	2.46	1.50	4.04
Not good/very poor	21.49%	41.09%	4.59	2.75	7.68
Smoking (ref non-smoker)	5.72%	10.14%	1.86	0.79	4.38
More than 14 weekly alcoholic drinks (ref = < 14)	11.44%	6.97%	0.58	0.32	1.04
Number of mental health care behaviours (range 0–9)	0.59 (1.00)	0.74 (1.10)	1.14	0.96	1.35
**COVID-19 infection variables**					
COVID-19 infection severity in first 2 weeks (ref asymptomatic)					
Mild	48.45%	23.35%	1.37	0.41	4.60
Moderate	41.37%	65.67%	4.50	1.42	14.23
Severe	1.32%	7.85%	16.85	4.16	68.26
Dominant strain in the UK at time of COVID-19 infection (ref original variant)^a^					
Alpha (1 November 2020 to 30 June 2021)	35.34%	47.44%	1.34	0.78	2.27
Delta (1 July to 30 November 2021)	47.74%	35.56%	0.74	0.41	1.33
**Socio-demographics**					
Female (ref male)	53.00%	43.62%	0.69	0.47	1.00
Age (ref 60+)					
45–59	30.41%	33.26%	1.04	0.67	1.61
30–44	20.60%	20.63%	0.95	0.54	1.68
18–29	11.42%	6.70%	0.56	0.22	1.41
Ethnic minority groups (ref White)	5.00%	11.46%	2.46	1.00	6.04
Education (ref degree or higher)					
A-levels or vocational	31.93%	29.57%	1.45	0.97	2.18
Up to GCSE	26.85%	44.09%	2.57	1.66	3.97
Low household income (<£30,000)	39.63%	47.11%	1.36	0.91	2.02
Employed (ref not employed)	59.32%	59.82%	1.02	0.68	1.53
Key worker	25.18%	25.06%	0.99	0.65	1.52
Crowded household	9.93%	17.78%	1.96	1.05	3.68
Living arrangement (ref alone)					
With others, not with children	55.77%	49.52%	0.89	0.53	1.50
With others, including children	26.79%	33.06%	1.24	0.70	2.20
Live in a rural area (ref urban)	18.77%	23.77%	1.35	0.84	2.18
**Pre-existing conditions**					
Long-term physical health condition (ref none)	40.11%	55.34%	1.85	1.25	2.74
Long-term mental health condition (ref none)	13.88%	16.19%	1.20	0.75	1.91

*Note*. Data were weighted to the proportions of gender, age, ethnicity, country, and education obtained from the Office for National Statistics. GCSE refers to General Certificate of Secondary Education. ^a^No infections occurred after 30 November 2021 in the study sample

In the fully adjusted model, compared to people who had had very good or good quality sleep prior to infection, those who reported average and not good or very poor sleep were 2.4–3.5 times as likely to have developed long COVID (average sleep: odds ratio [OR]: 2.44; 95% confidence interval [CI]: 1.44 to 4.12; not good/very poor sleep: OR: 3.53; 95% CI: 2.01 to 6.21) (Table 2).

**Table 2:** Logistic regressions predicting self-reported long COVID from health behaviours in the month prior to COVID-19 infection, weighted (*N* = 1581)

	Self-reported long COVID					
	OR	SE	T	P	95% CI	95% CI
**Model 1**						
Exercise frequency (ref no exercise)						
< 30 min to 2 hours	0.91	0.30	−0.29	0.77	0.47	1.74
3h hours	0.65	0.25	−1.11	0.27	0.31	1.38
Days of fresh air (> 15 minutes)	0.96	0.05	−0.72	0.47	0.87	1.07
Sleep quality (ref very good/good)						
Average	**2.39**	**0.59**	**3.54**	**< 0.01**	**1.47**	**3.86**
Not good/very poor	**4.13**	**1.14**	**5.11**	**< 0.01**	**2.40**	**7.11**
Smoking (ref non-smoker)	1.74	0.84	1.16	0.25	0.68	4.47
More than 14 weekly alcoholic drinks (ref = < 14)	0.60	0.18	−1.65	0.10	0.33	1.10
Number of mental health care behaviours	1.03	0.09	0.39	0.70	0.88	1.22
**Model 2**						
Exercise frequency (ref no exercise)						
< 30 min to 2 hours	0.99	0.33	−0.04	0.97	0.51	1.91
3h hours	0.71	0.28	−0.87	0.39	0.33	1.54
Days of fresh air (> 15 minutes)	0.98	0.06	−0.28	0.78	0.88	1.10
Sleep quality (ref very good/good)						
Average	**2.40**	**0.64**	**3.31**	**< 0.01**	**1.43**	**4.04**
Not good/very poor	**3.72**	**1.07**	**4.57**	**< 0.01**	**2.12**	**6.54**
Smoking (ref non-smoker)	2.19	1.22	1.40	0.16	0.73	6.54
More than 14 weekly alcoholic drinks (ref = < 14)	0.70	0.24	−1.02	0.31	0.36	1.38
Number of mental health care behaviours	0.97	0.08	−0.35	0.72	0.82	1.14
**Model 3**						
Exercise frequency (ref no exercise)						
< 30 min to 2 hours	1.02	0.36	0.06	0.95	0.51	2.05
3h hours	0.62	0.24	−1.22	0.22	0.28	1.34
Days of fresh air (> 15 minutes)	1.00	0.06	−0.05	0.96	0.89	1.12
Sleep quality (ref very good/good)						
Average	**2.43**	**0.64**	**3.35**	**< 0.01**	**1.44**	**4.08**
Not good/very poor	**3.54**	**1.02**	**4.41**	**< 0.01**	**2.02**	**6.21**
Smoking (ref non-smoker)	1.71	0.66	1.41	0.16	0.81	3.62
More than 14 weekly alcoholic drinks (ref = < 14)	0.72	0.27	−0.86	0.39	0.35	1.51
Number of mental health care behaviours	1.06	0.09	0.73	0.47	0.90	1.26
**Model 4**						
Exercise frequency (ref no exercise)						
< 30 min to 2 hours	0.95	0.34	−0.13	0.89	0.48	1.91
3h hours	0.57	0.23	−1.39	0.16	0.26	1.26
Days of fresh air (> 15 minutes)	1.00	0.06	−0.07	0.94	0.89	1.11
Sleep quality (ref very good/good)						
Average	**2.44**	**0.65**	**3.33**	**< 0.01**	**1.44**	**4.12**
Not good/very poor	**3.53**	**1.02**	**4.38**	**< 0.01**	**2.01**	**6.21**
Smoking (ref non-smoker)	1.72	0.64	1.47	0.14	0.83	3.57
More than 14 weekly alcoholic drinks (ref = < 14)	0.73	0.27	−0.83	0.40	0.35	1.53
Number of mental health care behaviours	1.10	0.10	1.13	0.26	0.93	1.31

*Note*. Data were weighted to the proportions of gender, age, ethnicity, country, and education obtained from the Office for National Statistics. Model 1 included only health behaviours (in the same model), Model 2 additionally adjusted for COVID-19 infection variables, Model 3 additionally adjusted for socio-demographic characteristics, and Model 4 additionally adjusted for pre-existing conditions

For individuals with long COVID (Table 3), more days of fresh air for at least 15 minutes reduced the likelihood of difficulty with mobility (OR: 0.85; 95% CI: 0.72 to 0.99; Table 4) but in the fully adjusted model (OR: 0.85; 95% CI: 0.71 to 1.00). Not good/very poor-quality sleep increased the likelihood of difficulty with cognition (OR: 3.16; 95% CI: 1.15 to 8.69) (Table 5), but not once covariates were included (OR: 3.06; 95% CI: 0.96 to 9.74). Having smoked in the month prior to infection with COVID-19 was associated with a more than eight-fold increased risk (OR: 8.39; 95% CI: 1.86 to 37.91; Table 6) of having difficulty with self-care, whilst weekly exercise of at least 3 hours reduced this likelihood (OR: 0.05; 95% CI: 0.01 to 0.39) in the fully adjusted model.

**Table 3:** Descriptive characteristics of participants with long COVID by specific long COVID symptom, weighted (*N* = 293)

	Difficulty with mobility *N* = 159	Difficulty with cognition *N* = 199	Difficulty with self-care *N* = 45
	Prop.	Prop.	Prop.
**Long COVID variables**			
Long COVID status			
Yes, but not formally diagnosed	73.42%	76.61%	62.14%
Yes, formally diagnosed	26.58%	23.39%	37.86%
Presence of specific long COVID symptoms			
Mobility (e.g., walking or climbing steps)	–	58.81%	100.00%
Cognition (e.g., remembering or concentrating)	66.33%	–	84.85%
Self-care (e.g., washing all over or dressing)	28.49%	21.44%	–
**Weekly health behaviours in month prior to COVID-19 infection**			
Exercise frequency (ref no exercise)			
< 30 min to 2 hours	65.22%	64.15%	66.67%
3h hours	20.29%	24.83%	10.37%
Days of fresh air (> 15 minutes)	4.89 (2.54)	5.19 (2.41)	4.38 (2.31)
Sleep quality (ref very good/good)			
Average	40.71%	41.26%	28.36%
Not good/very poor	46.33%	49.04%	62.21%
Smoking (ref non-smoker)	8.41%	13.26%	13.43%
More than 14 weekly alcoholic drinks (ref = < 14)	8.84%	7.44%	8.36%
Number of mental health care behaviours (range 0–9)	0.85 (1.26)	0.80 (1.11)	0.93 (1.12)
**COVID-19 infection variables**			
COVID-19 infection severity in first 2 weeks (ref asymptomatic)			
Mild	18.12%	16.68%	3.96%
Moderate	65.58%	71.58%	74.85%
Severe	11.83%	8.53%	13.99%
Dominant strain in the UK at time of COVID-19 infection (ref original variant)			
Alpha (1 November 2020 to 30 June 2021)	47.39%	51.48%	39.89%
Delta (1 July to 30 November 2021)	35.03%	37.98%	40.67%
Omicron (1 December 2021-)	0.00%	0.00%	0.00%
**Socio-demographics**			
Female (ref male)	39.48%	51.61%	46.72%
Age (ref 60+)			
45–59	31.89%	36.73%	32.44%
30–44	12.36%	22.66%	3.45%
18–29	1.21%	0.62%	0.00%
Ethnic minority groups (ref White)	4.91%	14.75%	8.97%
Education (ref degree or higher)			
A-levels or vocational	26.03%	26.08%	30.11%
Up to GCSE	51.14%	50.58%	50.34%
Low household income (<£30,000)	63.72%	47.78%	75.89%
Employed (ref not employed)	50.71%	66.19%	27.54%
Key worker	16.03%	27.71%	11.80%
Crowded household	13.65%	15.32%	14.30%
Living arrangement (ref alone) With others, not with children	52.90%	49.62%	50.55%
With others, including children	24.68%	33.01%	26.26%
Live in a rural area (ref urban)	23.19%	23.12%	24.38%
**Pre-existing conditions**			
Long-term physical health condition (ref none)	66.34%	57.66%	82.25%
Long-term mental health condition (ref none)	16.80%	18.78%	21.89%

*Note*. Data were weighted to the proportions of gender, age, ethnicity, country, and education obtained from the Office for National Statistics. GCSE refers to General Certificate of Secondary Education

**Table 4:** Logistic regressions predicting the development of difficulty with mobility from health behaviours in the month prior to COVID-19 infection, weighted (*N* = 293)

	Difficulty with mobility					
	OR	SE	T	P	95% CI	95% CI
**Model 1**						
Exercise frequency (ref no exercise)						
< 30 min to 2 hours	0.69	0.43	−0.59	0.55	0.20	2.35
3h hours	0.54	0.37	−0.91	0.37	0.14	2.06
Days of fresh air (> 15 minutes)	**0.84**	**0.07**	**−2.12**	**0.03**	**0.71**	**0.99**
Sleep quality (ref very good/good)						
Average	1.02	0.47	0.05	0.96	0.42	2.49
Not good/very poor	1.42	0.68	0.74	0.46	0.56	3.63
Smoking (ref non-smoker)	0.75	0.51	−0.42	0.68	0.20	2.82
More than 14 weekly alcoholic drinks (ref = < 14)	2.24	1.28	1.41	0.16	0.73	6.84
Number of mental health care behaviours	1.13	0.16	0.87	0.39	0.85	1.50
**Model 2**						
Exercise frequency (ref no exercise)						
< 30 min to 2 hours	0.61	0.42	−0.73	0.47	0.16	2.32
3h hours	0.47	0.35	−1.03	0.30	0.11	1.99
Days of fresh air (> 15 minutes)	**0.85**	**0.07**	**−2.06**	**0.04**	**0.72**	**0.99**
Sleep quality (ref very good/good)						
Average	1.16	0.55	0.30	0.76	0.45	2.95
Not good/very poor	1.47	0.73	0.78	0.44	0.56	3.90
Smoking (ref non-smoker)	0.79	0.54	−0.35	0.73	0.20	3.04
More than 14 weekly alcoholic drinks (ref = < 14)	2.46	1.55	1.43	0.15	0.71	8.47
Number of mental health care behaviours	1.09	0.17	0.54	0.59	0.80	1.47
**Model 3**						
Exercise frequency (ref no exercise)						
< 30 min to 2 hours	1.16	0.88	0.20	0.84	0.26	5.10
3h hours	0.60	0.49	−0.62	0.53	0.12	2.98
Days of fresh air (> 15 minutes)	0.85	0.07	−1.95	0.05	0.72	1.00
Sleep quality (ref very good/good)						
Average	1.28	0.69	0.47	0.64	0.45	3.67
Not good/very poor	1.46	0.78	0.72	0.47	0.52	4.14
Smoking (ref non-smoker)	1.07	0.69	0.11	0.91	0.30	3.78
More than 14 weekly alcoholic drinks (ref = < 14)	3.21	2.15	1.74	0.08	0.87	11.92
Number of mental health care behaviours	1.22	0.20	1.25	0.21	0.89	1.68
**Model 4**						
Exercise frequency (ref no exercise)						
< 30 min to 2 hours	1.06	0.78	0.08	0.93	0.25	4.47
3h hours	0.51	0.42	−0.83	0.41	0.10	2.53
Days of fresh air (> 15 minutes)	0.85	0.07	−1.91	0.06	0.71	1.00
Sleep quality (ref very good/good)						
Average	1.16	0.64	0.28	0.78	0.39	3.44
Not good/very poor	1.37	0.75	0.58	0.56	0.47	4.02
Smoking (ref non-smoker)	1.07	0.69	0.11	0.91	0.30	3.78
More than 14 weekly alcoholic drinks (ref = < 14)	3.53	2.45	1.81	0.07	0.90	13.78
Number of mental health care behaviours	1.37	0.26	1.64	0.10	0.94	1.98

*Note*. Data were weighted to the proportions of gender, age, ethnicity, country, and education obtained from the Office for National Statistics. Model 1 included only health behaviours (in the same model), Model 2 additionally adjusted for COVID-19 infection variables, Model 3 additionally adjusted for socio-demographic characteristics, and Model 4 additionally adjusted for pre-existing conditions

**Table 5:** Logistic regressions predicting the development of difficulty with cognition from health behaviours in the month prior to COVID-19 infection, weighted (*N* = 293)

	Difficulty with cognition					
	OR	SE	T	P	95% CI	95% CI
**Model 1**						
Exercise frequency (ref no exercise)						
< 30 min to 2 hours	1.54	0.98	0.68	0.50	0.44	5.38
3h hours	1.85	1.28	0.88	0.38	0.47	7.20
Days of fresh air (> 15 minutes)	0.92	0.09	−0.85	0.40	0.77	1.11
Sleep quality (ref very good/good)						
Average	1.27	0.59	0.52	0.60	0.51	3.15
Not good/very poor	**3.16**	**1.63**	**2.23**	**0.03**	**1.15**	**8.69**
Smoking (ref non-smoker)	3.27	2.68	1.45	0.15	0.66	16.30
More than 14 weekly alcoholic drinks (ref = < 14)	1.26	0.66	0.44	0.66	0.45	3.54
Number of mental health care behaviours	1.08	0.26	0.31	0.76	0.67	1.72
**Model 2**						
Exercise frequency (ref no exercise)						
< 30 min to 2 hours	1.18	0.79	0.25	0.80	0.32	4.36
3h hours	1.36	0.99	0.42	0.67	0.32	5.68
Days of fresh air (> 15 minutes)	0.92	0.08	−0.99	0.32	0.78	1.08
Sleep quality (ref very good/good)						
Average	1.20	0.53	0.40	0.69	0.50	2.84
Not good/very poor	2.63	1.32	1.92	0.05	0.98	7.03
Smoking (ref non-smoker)	4.03	4.14	1.36	0.18	0.54	30.18
More than 14 weekly alcoholic drinks (ref = < 14)	1.68	0.90	0.97	0.33	0.59	4.81
Number of mental health care behaviours	1.01	0.26	0.03	0.97	0.61	1.67
**Model 3**						
Exercise frequency (ref no exercise)						
< 30 min to 2 hours	0.94	0.79	−0.07	0.95	0.18	4.87
3h hours	0.88	0.78	−0.14	0.89	0.16	4.98
Days of fresh air (> 15 minutes)	0.91	0.08	−1.06	0.29	0.76	1.09
Sleep quality (ref very good/good)						
Average	1.05	0.57	0.09	0.93	0.36	3.06
Not good/very poor	3.09	1.81	1.92	0.05	0.98	9.76
Smoking (ref non-smoker)	2.50	1.96	1.17	0.24	0.54	11.65
More than 14 weekly alcoholic drinks (ref = < 14)	1.41	0.97	0.50	0.62	0.37	5.45
Number of mental health care behaviours	1.17	0.31	0.61	0.54	0.70	1.97
**Model 4**						
Exercise frequency (ref no exercise)						
< 30 min to 2 hours	0.94	0.78	−0.08	0.94	0.18	4.80
3h hours	0.87	0.77	−0.16	0.88	0.15	4.92
Days of fresh air (> 15 minutes)	0.90	0.08	−1.10	0.27	0.76	1.08
Sleep quality (ref very good/good)						
Average	1.02	0.57	0.04	0.97	0.35	3.02
Not good/very poor	3.06	1.81	1.89	0.06	0.96	9.74
Smoking (ref non-smoker)	2.46	1.91	1.16	0.25	0.54	11.30
More than 14 weekly alcoholic drinks (ref = < 14)	1.36	0.94	0.45	0.65	0.35	5.26
Number of mental health care behaviours	1.17	0.32	0.58	0.56	0.69	2.00

*Note*. Data were weighted to the proportions of gender, age, ethnicity, country, and education obtained from the Office for National Statistics. Model 1 included only health behaviours (in the same model), Model 2 additionally adjusted for COVID-19 infection variables, Model 3 additionally adjusted for socio-demographic characteristics, and Model 4 additionally adjusted for pre-existing conditions

**Table 6:** Logistic regressions predicting the development of difficulty with self-care from health behaviours in the month prior to COVID-19 infection, weighted (*N* = 293)

	Difficulty with self-care					
	OR	SE	T	P	95% CI	95% CI
**Model 1**						
Exercise frequency (ref no exercise)						
< 30 min to 2 hours	0.82	0.58	−0.28	0.78	0.20	3.30
3h hours	0.27	0.26	−1.33	0.18	0.04	1.85
Days of fresh air (> 15 minutes)	0.83	0.08	− 1.89	0.06	0.68	1.01
Sleep quality (ref very good/good)						
Average	0.94	0.65	−0.09	0.93	0.24	3.66
Not good/very poor	2.36	1.56	1.29	0.20	0.64	8.63
Smoking (ref non-smoker)	1.75	0.98	1.00	0.32	0.58	5.26
More than 14 weekly alcoholic drinks (ref = < 14)	1.39	0.85	0.55	0.59	0.42	4.61
Number of mental health care behaviours	0.97	0.17	−0.14	0.89	0.69	1.38
**Model 2**						
Exercise frequency (ref no exercise)						
< 30 min to 2 hours	0.78	0.64	−0.30	0.76	0.16	3.84
3h hours	0.25	0.28	−1.22	0.22	0.03	2.31
Days of fresh air (> 15 minutes)	0.82	0.09	−1.73	0.08	0.66	1.03
Sleep quality (ref very good/good)						
Average	0.93	0.75	−0.08	0.93	0.19	4.49
Not good/very poor	2.02	1.59	0.89	0.37	0.43	9.45
Smoking (ref non-smoker)	1.83	1.23	0.90	0.37	0.49	6.83
More than 14 weekly alcoholic drinks (ref = < 14)	1.70	1.20	0.75	0.45	0.43	6.80
Number of mental health care behaviours	0.89	0.16	−0.66	0.51	0.62	1.27
**Model 3**						
Exercise frequency (ref no exercise)						
< 30 min to 2 hours	0.99	0.79	−0.01	0.99	0.21	4.74
3h hours	0.16	0.15	−1.95	0.05	0.03	1.01
Days of fresh air (> 15 minutes)	0.84	0.09	−1.52	0.13	0.68	1.05
Sleep quality (ref very good/good)						
Average	0.54	0.45	−0.74	0.46	0.11	2.73
Not good/very poor	0.69	0.58	−0.44	0.66	0.13	3.56
Smoking (ref non-smoker)	**3.95**	**2.75**	**1.97**	**0.05**	**1.01**	**15.46**
More than 14 weekly alcoholic drinks (ref = < 14)	1.85	1.47	0.77	0.44	0.39	8.80
Number of mental health care behaviours	0.78	0.16	−1.17	0.24	0.52	1.18
**Model 4**						
Exercise frequency (ref no exercise)						
< 30 min to 2 hours	0.66	0.48	−0.56	0.57	0.16	2.78
3h hours	**0.05**	**0.05**	**−2.83**	**< 0.01**	**0.01**	**0.39**
Days of fresh air (> 15 minutes)	0.80	0.10	−1.85	0.06	0.63	1.01
Sleep quality (ref very good/good)						
Average	0.41	0.37	−0.99	0.32	0.07	2.36
Not good/very poor	0.71	0.66	−0.37	0.71	0.12	4.35
Smoking (ref non-smoker)	**8.39**	**6.45**	**2.76**	**0.01**	**1.86**	**37.91**
More than 14 weekly alcoholic drinks (ref = < 14)	1.01	0.81	0.01	0.99	0.21	4.88
Number of mental health care behaviours	0.86	0.23	−0.57	0.57	0.50	1.46

*Note*. Data were weighted to the proportions of gender, age, ethnicity, country, and education obtained from the Office for National Statistics. Model 1 included only health behaviours (in the same model), Model 2 additionally adjusted for COVID-19 infection variables, Model 3 additionally adjusted for socio-demographic characteristics, and Model 4 additionally adjusted for pre-existing conditions

Results from sensitivity analyses indicated similar findings, but with some minor exceptions (Supplemental Tables S10-S17). Smoking increased the likelihood of long COVID (OR: 1.90; 95% CI: 1.05 to 3.43), having engaged in more mental health care behaviours predicted difficulty with mobility amongst those with long COVID (OR: 1.38; 95% CI: 1.03 to 1.85; Supplemental Table S11), whilst consumption of more than 14 alcoholic drinks in a single week increased the likelihood of difficulty with self-care by 5.24 (95% CI: 1.34 to 19.58; Supplemental Table S13). Being slightly or very overweight was associated with 1.63 times greater odds (95% CI: 1.04 to 2.55) of long COVID (Supplemental Table S14). Not good/very poor-quality sleep and more mental health care behaviours predicted difficulty with cognition (Supplemental Table S16), and none of the health behaviours associated with the other long COVID symptoms.

## Discussion

This study explored the relationship between modifiable health behaviours in the month preceding COVID-19 infection and the risk of developing long COVID in a longitudinal study of UK adults. Notably, there was little evidence of associations, with no relationship found in unadjusted or adjusted models in exercise, fresh air, smoking, alcohol consumption or mental health care behaviours. The only association with long COVID that was found was with sleep, with poorer sleep in the month prior to infection associated with a 2.4–3.5-fold increase in risk of developing long COVID. Amongst participants who had developed long COVID, regular exercise was associated with 95% lower odds of developing difficulties with self-care, whilst smoking was associated with more than an 8-fold increase in risk of developing such difficulties.

Comparison with other research on pre-infection health behaviours is difficult, as the vast majority of this work has focused on health conditions such as obesity, asthma, and higher pre-pandemic levels of psychological distress [6–10], Several of the health behaviours we examined, such as not smoking, a healthy diet, regular physical activity, and consuming fewer than 14 alcoholic drinks per week are all inversely associated with all-cause mortality and longer lifespan [30]. Nevertheless, our null findings are congruent with other research showing that identifying who is most at risk for long COVID is difficult to determine, even with factors such as symptom severity sometimes showing inconsistent associations with long COVID development [4, 16–18].

However, we did find associations between poor sleep and subsequent long COVID. Whilst sleep disturbances have been commonly reported in people suffering from long COVID [31, 32], to our knowledge this is the first study to examine pre-infection sleep quality in relation to long COVID development. Lack of sleep can compromise both innate and adaptive immune function, making individuals more susceptible to infectious disease, and reduce the effectiveness of vaccine response [14, 15]. Therefore, it is possible that poor sleep places the body in a more vulnerable state for tackling COVID-19 infection. However, it is also possible that poor sleep is an indicator of other psychological stressors that could in fact be the cause of a heightened risk of developing long COVID [33]. Experiencing adversities as well as worrying about adversities also predicted lower sleep quality in the first months of the pandemic [34], suggesting a potential biobehavioural pathway from stress to long COVID via impaired sleep [35].

We also found that meeting weekly physical activity guidelines (at least 3 hours a week) reduced odds of self-care difficulties, but this was not found for less frequent physical activity (e.g., 30 mins – 2 hours a week). Our findings echo those from a study that focused on physical activity pre-pandemic and adverse COVID-19 outcomes including hospitalisation and mortality [19]. Regular physical activity plays a critical role in reducing risk for acquiring and death from infectious disease, strengthening the immune system, and enhancing vaccine response [13]. Exercise may therefore attenuate COVID-19 sequelae and persistent symptoms by moderating the inflammatory response [36]. However, the relationship between exercise and long COVID symptoms may be complex. Although exercise may improve symptoms of long COVID, long COVID symptoms can also be triggered by physical activity [37], and should therefore be titrated according to individual patient needs [1, 38]. In potential future pandemics, public health guidelines should include an emphasis on maintaining physical activity, as this could help to reduce long-term consequences of infection.

Finally, having smoked in the month prior to becoming infected with COVID-19 was by far the largest predictor of difficulties with self-care amongst adults with long COVID. Although smoking has been associated with increased likelihood of more severe COVID-19 outcomes [39], to our knowledge only one other study has examined smoking as a risk factor for long COVID. In a cross-sectional study designed to be representative of the adult population in England, the odds of persistent COVID-19 symptoms (12 weeks or more) from smoking (OR: 1.35) were higher than those of being overweight (OR: 1.16) [7]. Our study extends these findings by showing a temporal relationship between smoking and the development of specific long COVID symptoms. Although the prevalence of smokers in England decreased over the last decade, certain groups continue to be more likely to smoke: people with a mental health condition and those working in lower skilled occupations [22]. Lower socio-economic status and pre-existing mental health conditions have both been found to be risk factors for developing long COVID [7, 10, 33], underscoring the importance of smoking cessation particularly for vulnerable groups. Some long COVID management guidelines recommend not smoking to manage symptoms such as breathlessness [40], but this advice is currently only within a subsection of the National Health Service’s COVID recovery guidance for patients which advises people to avoid smoking or vaping near their oxygen tank at home [41].

This study has several strengths as well as limitations. A major strength is its longitudinal design, particularly the measurement of health behaviours prior to infection with COVID-19, the latter of which is random and cannot be predicted. However, due to data limitations, we were not able to include important health behaviours such as diet and nutrition, which are key behavioural risks for morbidity [22]. We also assessed a limited number of long COVID symptoms, and did not assess fatigue, which is often the most commonly reported [3]. Although well-stratified across major demographic groups, the study sample is also not representative of the general UK population, and results therefore cannot be generalised. Finally, multiple associations were tested in the analyses, and false discovery rate is therefore possible. The hypotheses in the current study should be tested on larger samples which are also representative of the general UK population.

## Conclusions

Our findings add to the dearth of research on health behaviours prior to infection with COVID-19 and the development of long COVID and suggest the importance of regular physical activity and smoking cessation, as early interventions to reduce the likelihood of long COVID. Poor quality sleep prior to infection with COVID-19 is also associated with the development of long COVID. More research on modifiable risk factors for long COVID is important, given that at the time of writing, the estimated proportion of the UK population experiencing long COVID is estimated to be 2.8% [5]. With the removal of free testing, it is important to promote public health messages to help people minimise their risk of developing long-term debilitating symptoms.

## Supplementary Information

**Additional file 1: Table S1.** Wording of study developed items. **Table S2.** Pattern of missing data in study sample (*N* = 1581). **Table S3.** Complete case analysis: logistic regressions predicting the development of long COVID from health behaviours in the month prior to COVID-19 infection, weighted (*N* = 1430). **Table S4.** Complete case analysis: logistic regressions predicting the development of difficulty with mobility from health behaviours in the month prior to COVID-19 infection, weighted (*N* = 264). **Table S5.** Complete case analysis: logistic regressions predicting the development of difficulty with cognition from health behaviours in the month prior to COVID-19 infection, weighted (*N* = 264). **Table S6.** Complete case analysis: logistic regressions predicting the development of difficulty with self-care from health behaviours in the month prior to COVID-19 infection, weighted (*N* = 264). **Table S7.** Characteristics of excluded and included participants, unweighted. **Table S8.** Number of weeks prior to COVID-19 infection in which health behaviours were measured (*N* = 1581). **Table S9.** Weighted and unweighted sample characteristics (*N* = 1581). **Table S10.** Sensitivity analysis: logistic regressions predicting self-reported long COVID from health behaviours, with participants who were ‘unsure’ whether they had had long COVID in the case group (*N* = 1581), weighted. **Table S11.** Sensitivity analysis: logistic regressions predicting the development of difficulty with mobility from health behaviours with participants who were ‘unsure’ whether they had had long COVID in the case group (*N* = 523), weighted. **Table S12.** Sensitivity analysis: logistic regressions predicting the development of difficulty with cognition from health behaviours, with participants who were ‘unsure’ whether they had had long COVID in the case group (*N* = 523), weighted. **Table S13.** Sensitivity analysis: logistic regressions predicting the development of difficulty with self-care from health behaviours, with participants who were ‘unsure’ whether they had had long COVID in the case group (*N* = 512), weighted. **Table S14.** Sensitivity analysis: logistic regressions predicting the development of long COVID from health behaviours, including overweight/obesity status (*N* = 1283) weighted. **Table S15.** Sensitivity analysis: logistic regressions predicting the development of difficulty with mobility from health behaviours, including overweight/obesity status (*N* = 234), weighted. **Table S16.** Sensitivity analysis: logistic regressions predicting the development of difficulty with cognition from health behaviours, including overweight/obesity status (*N* = 234) weighted. **Table S17.** Sensitivity analysis: logistic regressions predicting the development of difficulty with self-care from health behaviours, including overweight/obesity status (*N* = 225) weighted.

## Data Availability

The dataset analysed for the current study is not yet publicly available due to funder restrictions. However, the UCL COVID-19 Social Study documentation and codebook are available for download at https://osf.io/jm8ra/. Statistical code is available upon request from Elise Paul (e.paul@ucl.ac.uk).
