# Supplementary material for: Health behaviours the month prior to COVID-19 infection and the development of self-reported long COVID and specific long COVID symptoms: a longitudinal analysis of 1581 UK adults
Source: BMC Public Health. 2022 Sep 9;22:1716. doi: 10.1186/s12889-022-14123-7 (PMC9462889; doi:10.1186/s12889-022-14123-7)
Supplement: Supplementary file 1 — Additional file 1: Table S1. Wording of study developed items. Table S2. Pattern of missing data in study sample (N = 1581). Table S3. Complete case analysis: logistic regressions predicting the development of long COVID from health behaviours in the month prior to COVID-19 infection, weighted (N = 1430). Table S4. Complete case analysis: logistic regressions predicting the development of difficulty with mobility from health behaviours in the month prior to COVID-19 infection, weighted (N = 264). Table S5. Complete case analysis: logistic regressions predicting the development of difficulty with cognition from health behaviours in the month prior to COVID-19 infection, weighted (N = 264). Table S6. Complete case analysis: logistic regressions predicting the development of difficulty with self-care from health behaviours in the month prior to COVID-19 infection, weighted (N = 264). Table S7. Characteristics of excluded and included participants, unweighted. Table S8. Number of weeks prior to COVID-19 infection in which health behaviours were measured (N = 1581). Table S9. Weighted and unweighted sample characteristics (N = 1581). Table S10. Sensitivity analysis: logistic regressions predicting self-reported long COVID from health behaviours, with participants who were ‘unsure’ whether they had had long COVID in the case group (N = 1581), weighted. Table S11. Sensitivity analysis: logistic regressions predicting the development of difficulty with mobility from health behaviours with participants who were ‘unsure’ whether they had had long COVID in the case group (N = 523), weighted. Table S12. Sensitivity analysis: logistic regressions predicting the development of difficulty with cognition from health behaviours, with participants who were ‘unsure’ whether they had had long COVID in the case group (N = 523), weighted. Table S13. Sensitivity analysis: logistic regressions predicting the development of difficulty with self-care from health behaviours, with participants [file 12889_2022_14123_MOESM1_ESM.pdf]

Supplemental Materials

The three sampling approaches were as follows. First, convenience sampling was used, including promoting the study through existing networks and mailing lists (including large databases of adults who had previously consented to be involved in health research across the UK), print and digital media coverage, and social media. Second, more targeted recruitment was undertaken focusing on groups who were anticipated to be less likely to take part in the research via our first strategy, including (i) individuals from a low-income background, (ii) individuals with no or few educational qualifications, and (iii) individuals who were unemployed. Third, the study was promoted via partnerships with third sector organisations to vulnerable groups, including adults with pre-existing mental health conditions, older adults, carers, and people experiencing domestic violence or abuse.

Table S1. Wording of study developed items

Variable	Usage	Question wording	Response options	
COVID-19 infection	Sample selection	Overall, do you believe you have ever had Covid-19?	• Yes, confirmed by a positive Covid-19 test at the time I felt ill<br>• Yes, confirmed by a positive antibody test since that shows I have the antibodies to the virus<br>• Yes, suspected by a doctor but not tested<br>• Yes, my own suspicions<br>• No, not that I know of	
Long covid	Outcome	Do you consider yourself to have (or have had) "Long Covid"?	• Yes - a medical professional has formally diagnosed me with "Long Covid"<br>• Yes - I have not been formally diagnosed, but I consider myself to have "Long Covid"<br>• No, I do not consider myself to have "Long Covid"<br>• I am unsure	
Specific long COVID symptoms	Outcome	Has Covid-19 left you with any difficulties in your ability to function day-to-day in any of the following areas?	• Mobility (e.g., difficulty walking or climbing steps)<br>• Cognition (e.g., difficulty remembering or concentrating)<br>• Self-care (e.g., difficulty washing all over or dressing)	• No difficulty<br>• Some difficulty<br>• A lot of difficulty<br>• Unable to do
Exercise frequency	Health behaviour	Please answer the questions below FOCUSING ON A SINGLE DAY. This day should by THE LAST WEEK DAY (i.e. Monday to Friday). This may be YESTERDAY or it may be before the weekend. How much time did you spend on the last weekday doing the following?	• Exercise	• Did not do<br>• < 30 mins<br>• 30 mins-2 hours<br>• 3-5 hours<br>• 6-+ hours
Days of fresh air (> 15 min.)	Health behaviour	In the past 7 days, how many days have you: been outside for 15 minutes or more (including on a balcony or in the garden)?	Range: 0-7	
Sleep quality	Health behaviour	Over the past week, how has your sleep been?	• Very good<br>• Good	

			• Average<br>• Not good<br>• Very poor
Smoking	Health behaviour	Do you currently smoke?	• No<br>• Current light smoker (9 or less a day)<br>• Current moderate smoker (10-19 a day)<br>• Current heavy smoker (20+ a day)
More than 14 weekly alcoholic drinks (ref =< 14)	Health behaviour	How many alcoholic drinks have you had in the past week (e.g. how many glasses of wine / pints of beer or cider / shots of spirits)?	Range: 0-21+

Number of mental health care behaviours	Health behaviour	In the past week have you done any of the following to support your mental health? (Tick any that apply)	• Taken medication (e.g. anti-depressants)<br>• Spoken with a psychiatrist, psychologist or other mental health professional<br>• Spoken with a GP or other healthcare professional about your mental health<br>• Spoken to somebody on a support helpline (e.g. Samaritans or NHS Volunteers)<br>• Accessed an online mental health programme (e.g. CBT)<br>• Spoken with others on an online mental health forum<br>• Used other mental health resources (e.g. self-help books, videos, or apps)<br>• Spent time on self-care specifically to help your mental health (e.g. mindfulness, meditation, or planning time for hobbies or relaxation)<br>• Spoken about your mental health to a friend or family member<br>• None of the above
COVID-19 severity	Covariate	How severe were your symptoms in the first 1-2 weeks?	• I was hospitalised<br>• I experienced symptoms and had to rest in bed<br>• I experienced symptoms but was able to carry on with daily activities<br>• I was asymptomatic
Ethnicity	Covariate	What is your ethnicity?	• Asian/Asian British - Indian, Pakistani, Bangladeshi, other<br>• Black/Black British - Caribbean, African, other<br>• Mixed race - White and Black/Black British<br>• Mixed race – other<br>• White - British, Irish, other<br>• Chinese/Chinese British<br>• Middle Eastern/Middle Eastern British - Arab, Turkish, other<br>• Other ethnic group<br>• Prefer not to say

Table S2. Pattern of missing data in study sample (N = 1,581)

Variable	Proportion with missing data
Household income	8.01%
Employment status	0
Key worker status	0
Household crowding status	0
Living arrangement	0
Live in a rural area	0
COVID-19 infection severity	0.83%
Dominant strain in the UK at time of COVID-19 infection	0
Long-term physical health condition	0
Long-term mental health condition	0
Exercise frequency	0.11%
Number of days of fresh air	0.44%
Sleep quality	0
Smoking	0
More than 14 weekly alcoholic drinks (ref =< 14) consumed in a single week	0
Number of mental health care behaviours	0.66%

Table S3. Complete case analysis: logistic regressions predicting the development of long COVID from health behaviours in the month prior to COVID-19 infection, weighted (N = 1,430)

	Self-reported long COVID					
	OR	SE	T	P	95% CI	95% CI
**Model 1**						
Exercise frequency (ref no exercise)						
<30 min to 2 hours	0.93	0.31	-0.23	0.82	0.48	1.78
3+ hours	0.69	0.27	-0.95	0.34	0.32	1.49
Days of fresh air (> 15 minutes)	0.97	0.05	-0.62	0.54	0.87	1.08
Sleep quality (ref very good/good)						
Average	**2.26**	**0.58**	**3.14**	**<0.01**	**1.36**	**3.75**
Not good/very poor	**3.78**	**1.10**	**4.55**	**<0.01**	**2.13**	**6.70**
Smoking (ref non-smoker)	1.80	0.86	1.22	0.22	0.70	4.58
More than 14 weekly alcoholic drinks (ref =< 14)	0.61	0.19	-1.56	0.12	0.33	1.13
Number of mental health care behaviours	1.07	0.09	0.80	0.43	0.91	1.27
**Model 2**						
Exercise frequency (ref no exercise)						
<30 min to 2 hours	1.02	0.35	0.06	0.95	0.52	2.00
3+ hours	0.76	0.31	-0.69	0.49	0.34	1.67
Days of fresh air (> 15 minutes)	0.98	0.06	-0.34	0.74	0.87	1.10
Sleep quality (ref very good/good)						
Average	**2.29**	**0.64**	**2.98**	**<0.01**	**1.33**	**3.95**
Not good/very poor	**3.42**	**1.03**	**4.06**	**<0.01**	**1.89**	**6.18**
Smoking (ref non-smoker)	2.26	1.25	1.47	0.14	0.76	6.69
More than 14 weekly alcoholic drinks (ref =< 14)	0.69	0.24	-1.05	0.29	0.35	1.37
Number of mental health care behaviours	1.00	0.08	-0.06	0.95	0.84	1.18
**Model 3**						
Exercise frequency (ref no exercise)						
<30 min to 2 hours	1.11	0.40	0.28	0.78	0.54	2.26
3+ hours	0.65	0.27	-1.04	0.30	0.29	1.46
Days of fresh air (> 15 minutes)	0.99	0.06	-0.16	0.87	0.88	1.12
Sleep quality (ref very good/good)						
Average	**2.29**	**0.64**	**2.97**	**<0.01**	**1.33**	**3.97**
Not good/very poor	**3.25**	**1.00**	**3.85**	**<0.01**	**1.78**	**5.93**
Smoking (ref non-smoker)	1.71	0.65	1.43	0.15	0.82	3.60
More than 14 weekly alcoholic drinks (ref =< 14)	0.71	0.28	-0.87	0.38	0.33	1.52
Number of mental health care behaviours	1.10	0.10	1.04	0.30	0.92	1.31
**Model 4**						
Exercise frequency (ref no exercise)						
<30 min to 2 hours	1.06	0.38	0.17	0.87	0.52	2.16
3+ hours	0.62	0.26	-1.15	0.25	0.28	1.40
Days of fresh air (> 15 minutes)	0.99	0.06	-0.15	0.88	0.88	1.11
Sleep quality (ref very good/good)						
Average	**2.30**	**0.65**	**2.94**	**<0.01**	**1.32**	**4.01**
Not good/very poor	**3.20**	**0.99**	**3.77**	**<0.01**	**1.75**	**5.86**
Smoking (ref non-smoker)	1.72	0.63	1.48	0.14	0.84	3.55
More than 14 weekly alcoholic drinks (ref =< 14)	0.72	0.28	-0.86	0.39	0.33	1.53
Number of mental health care behaviours	1.11	0.10	1.11	0.27	0.92	1.33

Note. Data were weighted to the proportions of gender, age, ethnicity, country, and education obtained from the Office for National Statistics. Model 1 included only health behaviours (in the same model), Model 2 additionally adjusted for COVID-19 infection variables, Model 3 additionally adjusted for socio-demographic characteristics, and Model 4 additionally adjusted for pre-existing conditions.

Table S4. Complete case analysis: logistic regressions predicting the development of difficulty with mobility from health behaviours in the month prior to COVID-19 infection, weighted (N =264)

	Difficulty with mobility					
	OR	SE	T	P	95% CI	95% CI
**Model 1**						
Exercise frequency (ref no exercise)						
<30 min to 2 hours	0.60	0.38	-0.80	0.42	0.17	2.10
3+ hours	0.51	0.37	-0.93	0.35	0.13	2.08
Days of fresh air (> 15 minutes)	0.85	0.07	-1.82	0.07	0.72	1.01
Sleep quality (ref very good/good)						
Average	0.89	0.42	-0.24	0.81	0.35	2.26
Not good/very poor	1.17	0.59	0.31	0.76	0.44	3.13
Smoking	0.76	0.52	-0.40	0.69	0.20	2.88
More than 14 weekly alcoholic drinks (ref =< 14)	2.51	1.51	1.52	0.13	0.77	8.16
Number of mental health care behaviours	1.24	0.19	1.43	0.15	0.92	1.68
**Model 2**						
Exercise frequency (ref no exercise)						
<30 min to 2 hours	0.48	0.35	-0.99	0.32	0.11	2.03
3+ hours	0.45	0.35	-1.02	0.31	0.10	2.10
Days of fresh air (> 15 minutes)	0.87	0.08	-1.61	0.11	0.73	1.03
Sleep quality (ref very good/good)						
Average	1.10	0.54	0.19	0.85	0.42	2.89
Not good/very poor	1.26	0.66	0.44	0.66	0.45	3.49
Smoking (ref non-smoker)	0.77	0.53	-0.38	0.71	0.20	2.95
More than 14 weekly alcoholic drinks (ref =< 14)	2.93	1.86	1.69	0.09	0.84	10.16
Number of mental health care behaviours	1.20	0.19	1.12	0.26	0.87	1.64
**Model 3**						
Exercise frequency (ref no exercise)						
<30 min to 2 hours	1.08	0.86	0.10	0.92	0.23	5.11
3+ hours	0.63	0.53	-0.55	0.58	0.12	3.24
Days of fresh air (> 15 minutes)	0.86	0.08	-1.70	0.09	0.72	1.02
Sleep quality (ref very good/good)						
Average	1.23	0.68	0.38	0.71	0.42	3.62
Not good/very poor	1.40	0.76	0.62	0.53	0.48	4.07
Smoking (ref non-smoker)	1.12	0.69	0.18	0.85	0.33	3.77
More than 14 weekly alcoholic drinks (ref =< 14)	**3.92**	**2.71**	**1.97**	**0.05**	**1.01**	**15.22**
Number of mental health care behaviours	1.26	0.21	1.38	0.17	0.91	1.76
**Model 4**						
Exercise frequency (ref no exercise)						
<30 min to 2 hours	0.94	0.70	-0.08	0.94	0.22	4.06
3+ hours	0.52	0.43	-0.78	0.43	0.10	2.66
Days of fresh air (> 15 minutes)	0.86	0.08	-1.66	0.10	0.71	1.03
Sleep quality (ref very good/good)						
Average	1.19	0.67	0.31	0.76	0.39	3.59
Not good/very poor	1.39	0.78	0.59	0.55	0.46	4.18
Smoking (ref non-smoker)	1.12	0.72	0.18	0.85	0.32	3.93
More than 14 weekly alcoholic drinks (ref =< 14)	**4.58**	**3.32**	**2.09**	**0.04**	**1.10**	**19.00**
Number of mental health care behaviours	1.43	0.28	1.80	0.07	0.97	2.11

Note. Data were weighted to the proportions of gender, age, ethnicity, country, and education obtained from the Office for National Statistics. Model 1 included only health behaviours (in the same model), Model 2 additionally adjusted for COVID-19 infection variables, Model 3 additionally adjusted for socio-demographic characteristics, and Model 4 additionally adjusted for pre-existing conditions.

Table S5. Complete case analysis: logistic regressions predicting the development of difficulty with cognition from health behaviours in the month prior to COVID-19 infection, weighted (N = 264)

	Difficulty with cognition					
	OR	SE	T	P	95% CI	95% CI
**Model 1**						
Exercise frequency (ref no exercise)						
<30 min to 2 hours	1.31	0.79	0.45	0.65	0.40	4.27
3+ hours	1.80	1.25	0.84	0.40	0.46	7.03
Days of fresh air (> 15 minutes)	0.89	0.08	-1.26	0.21	0.74	1.07
Sleep quality (ref very good/good)						
Average	1.21	0.59	0.39	0.70	0.47	3.13
Not good/very poor	3.24	1.72	2.21	0.03	1.14	9.16
Smoking (ref non-smoker)	3.28	2.71	1.43	0.15	0.65	16.61
More than 14 weekly alcoholic drinks (ref =< 14)	1.13	0.61	0.22	0.83	0.39	3.26
Number of mental health care behaviours	1.06	0.26	0.23	0.82	0.65	1.71
**Model 2**						
Exercise frequency (ref no exercise)						
<30 min to 2 hours	1.08	0.70	0.11	0.91	0.30	3.88
3+ hours	1.53	1.13	0.57	0.57	0.36	6.54
Days of fresh air (> 15 minutes)	0.89	0.08	-1.33	0.18	0.74	1.06
Sleep quality (ref very good/good)						
Average	1.19	0.56	0.38	0.71	0.48	2.98
Not good/very poor	2.73	1.45	1.89	0.06	0.96	7.75
Smoking (ref non-smoker)	4.15	4.29	1.38	0.17	0.55	31.47
More than 14 weekly alcoholic drinks (ref =< 14)	1.59	0.89	0.83	0.41	0.53	4.77
Number of mental health care behaviours	0.97	0.27	-0.10	0.92	0.57	1.66
**Model 3**						
Exercise frequency (ref no exercise)						
<30 min to 2 hours	0.98	0.75	-0.03	0.98	0.22	4.37
3+ hours	1.15	1.00	0.16	0.87	0.21	6.35
Days of fresh air (> 15 minutes)	0.87	0.08	-1.42	0.16	0.73	1.05
Sleep quality (ref very good/good)						
Average	1.20	0.71	0.31	0.76	0.38	3.82
Not good/very poor	**4.23**	**2.79**	**2.19**	**0.03**	**1.17**	**15.38**
Smoking (ref non-smoker)	2.65	2.10	1.23	0.22	0.56	12.54
More than 14 weekly alcoholic drinks (ref =< 14)	1.11	0.79	0.14	0.89	0.27	4.49
Number of mental health care behaviours	1.09	0.28	0.35	0.73	0.66	1.00
**Model 4**						
Exercise frequency (ref no exercise)						
<30 min to 2 hours	0.97	0.75	-0.04	0.97	0.21	4.41
3+ hours	1.16	1.02	0.17	0.87	0.21	6.47
Days of fresh air (> 15 minutes)	0.88	0.08	-1.38	0.17	0.73	1.06
Sleep quality (ref very good/good)						
Average	1.24	0.73	0.37	0.71	0.39	3.92
Not good/very poor	**4.35**	**2.86**	**2.24**	**0.03**	**1.20**	**15.79**
Smoking (ref non-smoker)	2.71	2.15	1.26	0.21	0.58	12.78
More than 14 weekly alcoholic drinks (ref =< 14)	1.15	0.83	0.19	0.85	0.28	4.75
Number of mental health care behaviours	1.11	0.31	0.37	0.71	0.64	1.90

Note. Data were weighted to the proportions of gender, age, ethnicity, country, and education obtained from the Office for National Statistics. Model 1 included only health behaviours (in the same model), Model 2 additionally adjusted for COVID-19 infection variables, Model 3 additionally adjusted for socio-demographic characteristics, and Model 4 additionally adjusted for pre-existing conditions.

Table S6. Complete case analysis: logistic regressions predicting the development of difficulty with self-care from health behaviours in the month prior to COVID-19 infection, weighted (N = 264)

	Difficulty with self-care					
	OR	SE	T	P	95% CI	95% CI
**Model 1**						
Exercise frequency (ref no exercise)						
<30 min to 2 hours	0.61	0.41	-0.73	0.46	0.17	2.25
3+ hours	0.26	0.26	-1.37	0.17	0.04	1.79
Days of fresh air (> 15 minutes)	0.85	0.09	-1.56	0.12	0.70	1.04
Sleep quality (ref very good/good)						
Average	0.85	0.62	-0.22	0.83	0.20	3.57
Not good/very poor	2.07	1.37	1.09	0.27	0.56	7.58
Smoking (ref non-smoker)	1.73	0.95	0.99	0.32	0.59	5.09
More than 14 weekly alcoholic drinks (ref =< 14)	1.41	0.86	0.57	0.57	0.43	4.64
Number of mental health care behaviours	1.03	0.18	0.15	0.88	0.73	1.45
**Model 2**						
Exercise frequency (ref no exercise)						
<30 min to 2 hours	0.63	0.47	-0.62	0.54	0.15	2.72
3+ hours	0.26	0.29	-1.22	0.22	0.03	2.26
Days of fresh air (> 15 minutes)	0.84	0.10	-1.53	0.12	0.67	1.05
Sleep quality (ref very good/good)						
Average	1.00	0.83	0.00	1.00	0.20	5.09
Not good/very poor	2.13	1.72	0.94	0.35	0.44	10.38
Smoking (ref non-smoker)	1.89	1.25	0.96	0.34	0.52	6.89
More than 14 weekly alcoholic drinks (ref =< 14)	1.96	1.32	1.00	0.32	0.52	7.37
Number of mental health care behaviours	0.88	0.16	-0.70	0.48	0.62	1.25
**Model 3**						
Exercise frequency (ref no exercise)						
<30 min to 2 hours	1.49	1.07	0.56	0.58	0.36	6.11
3+ hours	**0.18**	**0.15**	**-2.10**	**0.04**	**0.04**	**0.89**
Days of fresh air (> 15 minutes)	0.82	0.10	-1.69	0.09	0.64	1.03
Sleep quality (ref very good/good)						
Average	0.59	0.45	-0.69	0.49	0.13	2.63
Not good/very poor	1.26	0.97	0.30	0.76	0.28	5.71
Smoking (ref non-smoker)	**4.76**	**3.09**	**2.40**	**0.02**	**1.33**	**17.01**
More than 14 weekly alcoholic drinks (ref =< 14)	1.35	1.01	0.39	0.69	0.31	5.88
Number of mental health care behaviours	0.72	0.17	-1.38	0.17	0.45	1.15
**Model 4**						
Exercise frequency (ref no exercise)						
<30 min to 2 hours	0.90	0.65	-0.14	0.89	0.22	3.73
3+ hours	**0.07**	**0.07**	**-2.88**	**<0.01**	**0.01**	**0.43**
Days of fresh air (> 15 minutes)	0.81	0.10	-1.72	0.09	0.63	1.03
Sleep quality (ref very good/good)						
Average	0.51	0.42	-0.82	0.41	0.10	2.58
Not good/very poor	1.09	0.95	0.10	0.92	0.20	5.97
Smoking (ref non-smoker)	**6.84**	**4.84**	**2.72**	**0.01**	**1.71**	**27.35**
More than 14 weekly alcoholic drinks (ref =< 14)	1.07	0.87	0.08	0.93	0.22	5.28
Number of mental health care behaviours	0.81	0.22	-0.79	0.43	0.47	1.38

Note. Data were weighted to the proportions of gender, age, ethnicity, country, and education obtained from the Office for National Statistics. Model 1 included only health behaviours (in the same model), Model 2 additionally adjusted for COVID-19 infection variables, Model 3 additionally adjusted for socio-demographic characteristics, and Model 4 additionally adjusted for pre-existing conditions.

Table S7. Characteristics of excluded and included participants, unweighted

		Excluded<br>N = 30,790<br>Prop.	Included<br>N = 1,581<br>Prop.
**Long COVID variables**			
Long COVID status			
	Unsure	16.44%	12.70%
	Yes, but not formally diagnosed	12.96%	12.31%
	Yes, formally diagnosed	3.54%	3.87%
Presence of specific long COVID symptoms (amongst participants with long COVID)			
	Mobility (e.g., walking or climbing steps)	43.43%	43.02%
	Cognition (e.g., remembering or concentrating)	60.54%	59.27%
	Self-care (e.g., washing all over or dressing)	17.55%	10.71%
**Health behaviours in month prior to COVID-19 infection**			
Exercise frequency (ref no exercise)			
	<30 min to 2 hours	56.09%	67.05%
	3+ hours	37.98%	25.30%
Days of fresh air (> 15 minutes)		6.79 (0.77)	5.78 (1.91)
Sleep quality (ref very good/good)			
	Average	20.77%	41.52%
	Not good/very poor	72.85%	24.96%
Smoking (ref non-smoker)		9.49%	5.91%
More than 14 weekly alcoholic drinks (ref =< 14)		31.61%	9.94%
Number of mental health care behaviours		1.51 (1.47)	0.74 (1.05)
**COVID-19 infection variables**			
COVID-19 infection severity in first two weeks (ref asymptomatic)			
	Mild	35.00%	38.27%
	Moderate	59.13%	51.77%
	Severe	1.94%	2.40%
Dominant strain in the UK at time of COVID-19 infection (ref original variant)			
	Alpha (1 November 2020 to 30 June 2021)	12.48%	41.58%
	Delta (1 July to 30 November 2021)	31.93%	42.19%
	Omicron (1 December 2021-)	0.47%	0.00%
**Socio-demographics**			
Female (ref male)		74.67%	75.59%
Age (ref 60+)	45-59	41.48%	40.70%
	30-44	34.16%	23.91%
	18-29	19.65%	5.96%
Ethnic minority groups (ref White)		4.71%	3.64%
Education (ref degree or higher)	A-levels or vocational	3.76%	17.01%
	Up to GCSE	17.18%	13.09%
Low household income (<£30,000)		14.44%	34.45%
Employed (ref not employed)		41.30%	67.59%
Key worker		57.46%	27.33%
Crowded household		20.92%	9.28%
Living arrangement (ref alone)	With others, not with children	8.03%	52.24%
	With others, including children	57.27%	31.36%
Live in a rural area (ref urban)		21.22%	20.04%
**Pre-existing health conditions**			
Long-term physical health condition (ref none)		43.56%	38.87%
Long-term mental health condition (ref none)		16.42%	15.24%

Table S8. Number of weeks prior to COVID-19 infection in which health behaviours were measured (N = 1,581)

	Prop.
2	26.06%
3	24.02%
4	24.68%
5	21.76%
6	3.48%

Table S9. Weighted and unweighted sample characteristics (N = 1,581)

		Unweighted Prop.	Weighted Prop.
**Long COVID variables**			
Long COVID status			
	Unsure	12.70%	12.04%
	Yes, but not formally diagnosed	12.31%	16.68%
	Yes, formally diagnosed	3.87%	3.80%
Presence of specific long COVID symptoms			
	Mobility (e.g., walking or climbing steps)	43.02%	44.92%
	Cognition (e.g., remembering or concentrating)	59.27%	54.23%
	Self-care (e.g., washing all over or dressing)	10.71%	12.10%
**Health behaviours in month prior to COVID-19 infection**			
Exercise frequency (ref no exercise)			
	<30 min to 2 hours	67.05%	63.39%
	3+ hours	25.30%	27.72%
Days of fresh air (> 15 minutes)		5.79 (1.91)	5.63 (2.07)
Sleep quality (ref very good/good)			
	Average	41.52%	43.80%
	Not good/very poor	26.95%	26.06%
Smoking (ref non-smoker)		5.91%	7.49%
More than 14 weekly alcoholic drinks (ref =< 14)		9.94%	10.56%
Number of mental health care behaviours		0.74 (1.05)	0.63 (1.01)
**COVID-19 infection variables**			
COVID-19 infection severity in first two weeks (ref asymptomatic)			
	Mild	38.27%	41.11%
	Moderate	51.77%	48.22%
	Severe	2.40%	2.95%
Dominant strain in the UK at time of COVID-19 infection (ref original variant)			
	Alpha (1 November 2020 to 30 June 2021)	41.58%	38.36%
	Delta (1 July to 30 November 2021)	42.19%	45.04%
**Socio-demographics**			
Female (ref male)		75.59%	51.82%
Age (ref 60+)	45-59	40.70%	31.76%
	30-44	23.91%	20.36%
	18-29	5.96%	10.67%
Ethnic minority groups (ref White)		3.64%	6.12%
Education (ref degree or higher)	A-levels or vocational	17.01%	31.97%
	Up to GCSE	13.09%	30.68%
Low household income (<£30,000)		34.54%	42.55%
Employed (ref not employed)		67.59%	59.42%
Key worker		27.33%	24.94%
Crowded household		9.28%	11.64%
Living arrangement (ref alone)	With others, not with children	52.24%	54.69%
	With others, including children	31.36%	27.94%
Live in a rural area (ref urban)		20.04%	20.11%
**Pre-existing conditions**			
Long-term physical health condition (ref none)		38.87%	43.97%
Long-term mental health condition (ref none)		15.24%	14.96%

Note. Data in the weighted sample were weighted to the proportions of gender, age, ethnicity, country, and education obtained from the Office for National Statistics. GCSE refers to General Certificate of Secondary Education.

Table S10. Sensitivity analysis: logistic regressions predicting self-reported long COVID from health behaviours, with participants who were 'unsure' whether they had had long COVID in the case group (N = 1,581), weighted

	Self-reported long COVID					
	OR	SE	T	P	95% CI	95% CI
**Model 1**						
Exercise frequency (ref no exercise)						
<30 min to 2 hours	0.75	0.22	-0.97	0.33	0.42	1.35
3+ hours	0.54	0.18	-1.84	0.07	0.28	1.04
Days of fresh air (> 15 minutes)	0.98	0.04	-0.42	0.67	0.90	1.07
Sleep quality (ref very good/good)						
Average	**1.86**	**0.35**	**3.31**	**<0.01**	**1.29**	**2.69**
Not good/very poor	**2.63**	**0.57**	**4.48**	**<0.01**	**1.72**	**4.01**
Smoking (ref non-smoker)	**2.08**	**0.74**	**2.07**	**0.04**	**1.04**	**4.17**
More than 14 weekly alcoholic drinks (ref =< 14)	0.74	0.18	-1.24	0.22	0.45	1.20
Number of mental health care behaviours	1.04	0.07	0.61	0.54	0.91	1.20
**Model 2**						
Exercise frequency (ref no exercise)						
<30 min to 2 hours	0.68	0.23	-1.14	0.25	0.35	1.32
3+ hours	0.49	0.18	-1.95	0.05	0.24	1.00
Days of fresh air (> 15 minutes)	1.01	0.05	0.22	0.83	0.92	1.11
Sleep quality (ref very good/good)						
Average	**1.88**	**0.38**	**3.17**	**<0.01**	**1.27**	**2.78**
Not good/very poor	**2.44**	**0.53**	**4.09**	**<0.01**	**1.59**	**3.74**
Smoking (ref non-smoker)	**2.33**	**0.90**	**2.18**	**0.03**	**1.09**	**4.99**
More than 14 weekly alcoholic drinks (ref =< 14)	0.84	0.22	-0.67	0.50	0.49	1.41
Number of mental health care behaviours	0.98	0.07	-0.29	0.77	0.85	1.12
**Model 3**						
Exercise frequency (ref no exercise)						
<30 min to 2 hours	0.72	0.23	-1.02	0.31	0.38	1.36
3+ hours	**0.48**	**0.17**	**-2.05**	**0.04**	**0.24**	**0.97**
Days of fresh air (> 15 minutes)	1.03	0.05	0.57	0.57	0.94	1.13
Sleep quality (ref very good/good)						
Average	**1.84**	**0.37**	**3.08**	**<0.01**	**1.25**	**2.72**
Not good/very poor	**2.28**	**0.50**	**3.75**	**<0.01**	**1.48**	**3.51**
Smoking (ref non-smoker)	**1.95**	**0.61**	**2.13**	**0.03**	**1.06**	**3.61**
More than 14 weekly alcoholic drinks (ref =< 14)	0.83	0.23	-0.67	0.51	0.48	1.44
Number of mental health care behaviours	1.02	0.07	0.33	0.74	0.89	1.18
**Model 4**						
Exercise frequency (ref no exercise)						
<30 min to 2 hours	0.69	0.23	-1.11	0.27	0.36	1.33
3+ hours	**0.47**	**0.17**	**-2.09**	**0.04**	**0.23**	**0.95**
Days of fresh air (> 15 minutes)	1.03	0.05	0.59	0.55	0.94	1.13
Sleep quality (ref very good/good)						
Average	**1.84**	**0.37**	**3.08**	**<0.01**	**1.25**	**2.72**
Not good/very poor	**2.29**	**0.51**	**3.77**	**<0.01**	**1.49**	**3.54**
Smoking (ref non-smoker)	**1.90**	**0.57**	**2.12**	**0.03**	**1.05**	**3.43**
More than 14 weekly alcoholic drinks (ref =< 14)	0.82	0.23	-0.71	0.48	0.48	1.41
Number of mental health care behaviours	1.04	0.08	0.56	0.58	0.90	1.21

Note. Data were weighted to the proportions of gender, age, ethnicity, country, and education obtained from the Office for National Statistics. Model 1 included only health behaviours (in the same model), Model 2 additionally adjusted for COVID-19 infection variables, Model 3 additionally adjusted for socio-demographic characteristics, and Model 4 additionally adjusted for pre-existing conditions.

Table S11. Sensitivity analysis: logistic regressions predicting the development of difficulty with mobility from health behaviours with participants who were ‘unsure’ whether they had had long COVID in the case group (N = 523), weighted

	Difficulty with mobility					
	OR	SE	T	P	95% CI	95% CI
**Model 1**						
Exercise frequency (ref no exercise)						
<30 min to 2 hours	1.28	0.59	0.53	0.59	0.52	3.16
3+ hours	0.99	0.51	-0.02	0.99	0.36	2.71
Days of fresh air (> 15 minutes)	0.90	0.06	-1.53	0.13	0.79	1.03
Sleep quality (ref very good/good)						
Average	1.33	0.44	0.86	0.39	0.70	2.53
Not good/very poor	1.64	0.60	1.36	0.17	0.80	3.36
Smoking (ref non-smoker)	0.53	0.26	-1.28	0.20	0.21	1.39
More than 14 weekly alcoholic drinks (ref =< 14)	**2.33**	**1.00**	**1.96**	**0.05**	**1.00**	**5.42**
Number of mental health care behaviours	1.18	0.15	1.36	0.17	0.93	1.50
**Model 2**						
Exercise frequency (ref no exercise)						
<30 min to 2 hours	1.23	0.58	0.44	0.66	0.49	3.11
3+ hours	0.93	0.49	-0.13	0.90	0.33	2.60
Days of fresh air (> 15 minutes)	0.92	0.06	-1.21	0.23	0.80	1.05
Sleep quality (ref very good/good)						
Average	1.35	0.48	0.85	0.40	0.68	2.70
Not good/very poor	1.63	0.63	1.26	0.21	0.76	3.48
Smoking (ref non-smoker)	0.57	0.28	-1.14	0.25	0.21	1.51
More than 14 weekly alcoholic drinks (ref =< 14)	**2.66**	**1.28**	**2.04**	**0.04**	**1.04**	**6.81**
Number of mental health care behaviours	1.14	0.15	0.98	0.33	0.88	1.47
**Model 3**						
Exercise frequency (ref no exercise)						
<30 min to 2 hours	1.08	0.48	0.18	0.86	0.45	2.60
3+ hours	0.68	0.35	-0.76	0.45	0.25	1.85
Days of fresh air (> 15 minutes)	0.96	0.06	-0.56	0.58	0.85	1.10
Sleep quality (ref very good/good)						
Average	1.32	0.47	0.78	0.44	0.66	2.66
Not good/very poor	1.51	0.61	1.01	0.31	0.68	3.34
Smoking (ref non-smoker)	0.64	0.34	-0.84	0.40	0.22	1.83
More than 14 weekly alcoholic drinks (ref =< 14)	**2.80**	**1.45**	**1.99**	**0.05**	**1.01**	**7.72**
Number of mental health care behaviours	1.29	0.18	1.87	0.06	0.99	1.69
**Model 4**						
Exercise frequency (ref no exercise)						
<30 min to 2 hours	0.96	0.44	-0.09	0.93	0.39	2.37
3+ hours	0.61	0.32	-0.94	0.35	0.22	1.70
Days of fresh air (> 15 minutes)	0.97	0.07	-0.48	0.63	0.85	1.10
Sleep quality (ref very good/good)						
Average	1.26	0.45	0.65	0.52	0.63	2.54
Not good/very poor	1.52	0.62	1.01	0.31	0.68	3.40
Smoking (ref non-smoker)	0.59	0.33	-0.95	0.34	0.20	1.77
More than 14 weekly alcoholic drinks (ref =< 14)	2.60	1.34	1.85	0.06	0.94	7.15
Number of mental health care behaviours	**1.38**	**0.20**	**2.18**	**0.03**	**1.03**	**1.85**

Note. Data were weighted to the proportions of gender, age, ethnicity, country, and education obtained from the Office for National Statistics. Model 1 included only health behaviours (in the same model), Model 2 additionally adjusted for COVID-19 infection variables, Model 3 additionally adjusted for socio-demographic characteristics, and Model 4 additionally adjusted for pre-existing conditions.

Table S12. Sensitivity analysis: logistic regressions predicting the development of difficulty with cognition from health behaviours, with participants who were 'unsure' whether they had had long COVID in the case group (N = 523), weighted

	Difficulty with cognition					
	OR	SE	T	P	95% CI	95% CI
**Model 1**						
Exercise frequency (ref no exercise)						
<30 min to 2 hours	2.28	1.00	1.88	0.06	0.96	5.39
3+ hours	2.40	1.19	1.77	0.08	0.91	6.32
Days of fresh air (> 15 minutes)	0.96	0.07	-0.62	0.54	0.84	1.10
Sleep quality (ref very good/good)						
Average	1.18	0.38	0.53	0.60	0.63	2.22
Not good/very poor	**2.60**	**0.95**	**2.62**	**0.01**	**1.27**	**5.32**
Smoking (ref non-smoker)	1.26	0.70	0.41	0.68	0.42	3.77
More than 14 weekly alcoholic drinks (ref =< 14)	1.11	0.49	0.24	0.81	0.47	2.65
Number of mental health care behaviours	1.27	0.25	1.23	0.22	0.87	1.86
**Model 2**						
Exercise frequency (ref no exercise)						
<30 min to 2 hours	1.88	0.83	1.43	0.15	0.79	4.45
3+ hours	1.94	0.98	1.31	0.19	0.72	5.23
Days of fresh air (> 15 minutes)	0.97	0.07	-0.45	0.66	0.85	1.11
Sleep quality (ref very good/good)						
Average	1.07	0.35	0.21	0.84	0.57	2.03
Not good/very poor	**2.28**	**0.86**	**2.20**	**0.03**	**1.09**	**4.77**
Smoking (ref non-smoker)	1.39	0.89	0.51	0.61	0.40	4.86
More than 14 weekly alcoholic drinks (ref =< 14)	1.29	0.66	0.50	0.62	0.47	3.52
Number of mental health care behaviours	1.22	0.25	0.96	0.34	0.81	1.82
**Model 3**						
Exercise frequency (ref no exercise)						
<30 min to 2 hours	1.59	0.78	0.95	0.34	0.61	4.15
3+ hours	1.47	0.79	0.72	0.47	0.51	4.20
Days of fresh air (> 15 minutes)	0.95	0.07	-0.69	0.49	0.83	1.10
Sleep quality (ref very good/good)						
Average	1.12	0.37	0.33	0.74	0.58	2.15
Not good/very poor	2.14	0.85	1.91	0.06	0.98	4.67
Smoking (ref non-smoker)	1.27	0.62	0.49	0.62	0.49	3.30
More than 14 weekly alcoholic drinks (ref =< 14)	1.46	0.72	0.76	0.45	0.55	3.83
Number of mental health care behaviours	1.38	0.29	1.51	0.13	0.91	2.09
**Model 4**						
Exercise frequency (ref no exercise)						
<30 min to 2 hours	1.61	0.80	0.97	0.33	0.61	4.26
3+ hours	1.49	0.81	0.74	0.46	0.52	4.31
Days of fresh air (> 15 minutes)	0.96	0.07	-0.62	0.53	0.83	1.10
Sleep quality (ref very good/good)						
Average	1.11	0.37	0.31	0.76	0.57	2.15
Not good/very poor	2.14	0.84	1.94	0.05	0.99	4.61
Smoking (ref non-smoker)	1.24	0.61	0.43	0.66	0.47	3.24
More than 14 weekly alcoholic drinks (ref =< 14)	1.40	0.69	0.67	0.50	0.53	3.69
Number of mental health care behaviours	1.33	0.29	1.32	0.19	0.87	2.05

Note. Data were weighted to the proportions of gender, age, ethnicity, country, and education obtained from the Office for National Statistics. Model 1 included only health behaviours (in the same model), Model 2 additionally adjusted for COVID-19 infection variables, Model 3 additionally adjusted for socio-demographic characteristics, and Model 4 additionally adjusted for pre-existing conditions.

Table S13. Sensitivity analysis: logistic regressions predicting the development of difficulty with self-care from health behaviours, with participants who were 'unsure' whether they had had long COVID in the case group (N = 512), weighted

	Difficulty with self-care					
	OR	SE	T	P	95% CI	95% CI
**Model 1**						
Exercise frequency (ref no exercise)						
<30 min to 2 hours	0.88	0.51	-0.22	0.83	0.28	2.73
3+ hours	0.48	0.36	-0.98	0.33	0.11	2.08
Days of fresh air (> 15 minutes)	0.85	0.07	-1.93	0.05	0.72	1.00
Sleep quality (ref very good/good)						
Average	1.94	1.11	1.16	0.24	0.63	5.95
Not good/very poor	**3.46**	**1.97**	**2.18**	**0.03**	**1.13**	**10.56**
Smoking (ref non-smoker)	1.40	0.73	0.64	0.52	0.50	3.87
More than 14 weekly alcoholic drinks (ref =< 14)	**3.21**	**1.90**	**1.97**	**0.05**	**1.00**	**10.24**
Number of mental health care behaviours	1.09	0.18	0.51	0.61	0.79	1.49
**Model 2**						
Exercise frequency (ref no exercise)						
<30 min to 2 hours	0.95	0.69	-0.07	0.95	0.23	3.97
3+ hours	0.49	0.43	-0.80	0.42	0.09	2.75
Days of fresh air (> 15 minutes)	0.85	0.08	-1.73	0.08	0.71	1.02
Sleep quality (ref very good/good)						
Average	1.77	1.14	0.89	0.38	0.50	6.26
Not good/very poor	3.28	2.23	1.75	0.08	0.87	12.41
Smoking (ref non-smoker)	1.39	0.80	0.57	0.57	0.45	4.32
More than 14 weekly alcoholic drinks (ref =< 14)	**3.52**	**2.25**	**1.97**	**0.05**	**1.01**	**12.31**
Number of mental health care behaviours	1.01	0.17	0.08	0.94	0.73	1.41
**Model 3**						
Exercise frequency (ref no exercise)						
<30 min to 2 hours	0.75	0.51	-0.43	0.67	0.20	2.81
3+ hours	0.32	0.23	-1.55	0.12	0.08	1.35
Days of fresh air (> 15 minutes)	0.89	0.08	-1.24	0.22	0.74	1.07
Sleep quality (ref very good/good)						
Average	1.62	1.24	0.64	0.52	0.37	7.21
Not good/very poor	2.24	1.81	1.00	0.32	0.46	10.90
Smoking (ref non-smoker)	2.45	1.39	1.58	0.11	0.81	7.45
More than 14 weekly alcoholic drinks (ref =< 14)	**5.90**	**3.77**	**2.78**	**0.01**	**1.68**	**20.66**
Number of mental health care behaviours	1.01	0.20	0.07	0.94	0.68	1.51
**Model 4**						
Exercise frequency (ref no exercise)						
<30 min to 2 hours	0.46	0.29	-1.23	0.22	0.13	1.58
3+ hours	**0.18**	**0.13**	**-2.33**	**0.02**	**0.04**	**0.76**
Days of fresh air (> 15 minutes)	0.88	0.09	-1.29	0.20	0.72	1.07
Sleep quality (ref very good/good)						
Average	1.39	1.13	0.41	0.68	0.28	6.82
Not good/very poor	2.65	2.25	1.14	0.25	0.50	14.05
Smoking (ref non-smoker)	2.84	1.76	1.68	0.09	0.84	9.59
More than 14 weekly alcoholic drinks (ref =< 14)	**5.24**	**3.52**	**2.46**	**0.01**	**1.40**	**19.58**
Number of mental health care behaviours	1.12	0.26	0.47	0.64	0.71	1.75

Note. Data were weighted to the proportions of gender, age, ethnicity, country, and education obtained from the Office for National Statistics. Model 1 included only health behaviours (in the same model), Model 2 additionally adjusted for COVID-19 infection variables, Model 3 additionally adjusted for socio-demographic characteristics, and Model 4 additionally adjusted for pre-existing conditions.

Table S14. Sensitivity analysis: logistic regressions predicting the development of long COVID from health behaviours, including overweight/obesity status (N = 1,283) weighted

	Self-reported long COVID					
	OR	SE	T	P	95% CI	95% CI
**Model 1**						
Exercise frequency (ref no exercise)						
<30 min to 2 hours	0.89	0.33	-0.32	0.75	0.43	1.84
3+ hours	0.62	0.27	-1.10	0.27	0.27	1.44
Days of fresh air (> 15 minutes)	0.95	0.06	-0.83	0.41	0.85	1.07
Sleep quality (ref very good/good)						
Average	**2.25**	**0.61**	**2.99**	**<0.001**	**1.32**	**3.83**
Not good/very poor	**3.71**	**1.16**	**4.20**	**<0.001**	**2.01**	**6.84**
Smoking (ref non-smoker)	1.70	0.92	0.98	0.33	0.59	4.90
More than 14 weekly alcoholic drinks (ref =< 14)	0.85	0.28	-0.48	0.63	0.44	1.64
Number of mental health care behaviours	0.99	0.09	-0.11	0.92	0.83	1.18
Slightly/very overweight (ref under/normal weight)	**1.94**	**0.47**	**2.75**	**0.01**	**1.21**	**3.12**
**Model 2**						
Exercise frequency (ref no exercise)						
<30 min to 2 hours	0.99	0.39	-0.02	0.98	0.46	2.13
3+ hours	0.75	0.33	-0.65	0.52	0.32	1.78
Days of fresh air (> 15 minutes)	0.97	0.06	-0.57	0.57	0.85	1.09
Sleep quality (ref very good/good)						
Average	**2.09**	**0.63**	**2.44**	**0.01**	**1.16**	**3.77**
Not good/very poor	**3.05**	**0.99**	**3.44**	**<0.001**	**1.62**	**5.77**
Smoking (ref non-smoker)	2.48	1.57	1.44	0.15	0.72	8.54
More than 14 weekly alcoholic drinks (ref =< 14)	1.17	0.45	0.39	0.69	0.54	2.50
Number of mental health care behaviours	0.93	0.09	-0.79	0.43	0.78	1.11
Slightly/very overweight (ref under/normal weight)	**1.97**	**0.49**	**2.73**	**0.01**	**1.21**	**3.19**
**Model 3**						
Exercise frequency (ref no exercise)						
<30 min to 2 hours	1.10	0.44	0.24	0.81	0.50	2.42
3+ hours	0.65	0.28	-1.02	0.31	0.28	1.50
Days of fresh air (> 15 minutes)	0.98	0.06	-0.37	0.71	0.87	1.10
Sleep quality (ref very good/good)						
Average	**2.20**	**0.62**	**2.78**	**0.01**	**1.26**	**3.83**
Not good/very poor	**3.05**	**0.95**	**3.57**	**<0.001**	**1.65**	**5.62**
Smoking (ref non-smoker)	1.81	0.76	1.43	0.15	0.80	4.10
More than 14 weekly alcoholic drinks (ref =< 14)	1.27	0.52	0.58	0.56	0.57	2.85
Number of mental health care behaviours	1.03	0.10	0.30	0.76	0.85	1.24
Slightly/very overweight (ref under/normal weight)	**1.78**	**0.41**	**2.51**	**0.01**	**1.13**	**2.78**
**Model 4**						
Exercise frequency (ref no exercise)						
<30 min to 2 hours	1.08	0.44	0.19	0.85	0.49	2.38
3+ hours	0.63	0.28	-1.05	0.29	0.27	1.50
Days of fresh air (> 15 minutes)	0.97	0.06	-0.43	0.67	0.86	1.10
Sleep quality (ref very good/good)						
Average	**2.25**	**0.65**	**2.78**	**0.01**	**1.27**	**3.97**
Not good/very poor	**3.00**	**0.94**	**3.50**	**<0.001**	**1.62**	**5.56**
Smoking (ref non-smoker)	1.82	0.73	1.49	0.14	0.83	4.01
More than 14 weekly alcoholic drinks (ref =< 14)	1.31	0.54	0.66	0.51	0.59	2.94
Number of mental health care behaviours	1.03	0.11	0.27	0.79	0.84	1.26
Slightly/very overweight (ref under/normal weight)	**1.63**	**0.37**	**2.13**	**0.03**	**1.04**	**2.55**

Note. Data were weighted to the proportions of gender, age, ethnicity, country, and education obtained from the Office for National Statistics. Model 1 included only health behaviours (in the same model), Model 2 additionally adjusted for COVID-19 infection variables, Model 3 additionally adjusted for socio-demographic characteristics, and Model 4 additionally adjusted for pre-existing conditions.

Table S15. Sensitivity analysis: logistic regressions predicting the development of difficulty with mobility from health behaviours, including overweight/obesity status (N = 234), weighted

	Difficulty with mobility					
	OR	SE	T	P	95% CI	95% CI
**Model 1**						
Exercise frequency (ref no exercise)						
<30 min to 2 hours	0.75	0.49	-0.44	0.66	0.21	2.72
3+ hours	0.64	0.48	-0.59	0.55	0.15	2.78
Days of fresh air (> 15 minutes)	0.88	0.08	-1.43	0.15	0.74	1.05
Sleep quality (ref very good/good)						
Average	0.66	0.35	-0.78	0.44	0.23	1.87
Not good/very poor	0.99	0.55	-0.02	0.98	0.33	2.93
Smoking (ref non-smoker)	0.51	0.38	-0.91	0.37	0.12	2.19
More than 14 weekly alcoholic drinks (ref =< 14)	1.78	1.10	0.93	0.35	0.53	5.98
Number of mental health care behaviours	1.09	0.18	0.51	0.61	0.78	1.51
Slightly/very overweight (ref under/normal weight)	1.40	0.63	0.75	0.46	0.58	3.38
**Model 2**						
Exercise frequency (ref no exercise)						
<30 min to 2 hours	0.69	0.48	-0.53	0.59	0.18	2.71
3+ hours	0.67	0.54	-0.50	0.61	0.14	3.21
Days of fresh air (> 15 minutes)	0.90	0.08	-1.12	0.26	0.76	1.08
Sleep quality (ref very good/good)						
Average	0.63	0.31	-0.92	0.36	0.24	1.68
Not good/very poor	0.84	0.46	-0.32	0.75	0.29	2.47
Smoking (ref non-smoker)	0.74	0.52	-0.44	0.66	0.19	2.92
More than 14 weekly alcoholic drinks (ref =< 14)	2.52	1.88	1.24	0.22	0.58	10.91
Number of mental health care behaviours	1.06	0.18	0.31	0.75	0.75	1.49
Slightly/very overweight (ref under/normal weight)	1.27	0.57	0.54	0.59	0.53	3.05
**Model 3**						
Exercise frequency (ref no exercise)						
<30 min to 2 hours	1.32	1.07	0.34	0.73	0.27	6.50
3+ hours	0.74	0.69	-0.32	0.75	0.12	4.61
Days of fresh air (> 15 minutes)	0.90	0.09	-1.02	0.31	0.73	1.10
Sleep quality (ref very good/good)						
Average	0.77	0.47	-0.43	0.66	0.23	2.56
Not good/very poor	0.65	0.41	-0.69	0.49	0.19	2.23
Smoking (ref non-smoker)	0.88	0.62	-0.18	0.86	0.22	3.49
More than 14 weekly alcoholic drinks (ref =< 14)	4.45	3.46	1.92	0.06	0.97	20.45
Number of mental health care behaviours	1.29	0.24	1.36	0.17	0.89	1.87
Slightly/very overweight (ref under/normal weight)	1.46	0.68	0.80	0.42	0.58	3.66
**Model 4**						
Exercise frequency (ref no exercise)						
<30 min to 2 hours	1.33	1.03	0.37	0.71	0.29	6.10
3+ hours	0.73	0.68	-0.34	0.74	0.12	4.51
Days of fresh air (> 15 minutes)	0.90	0.10	-1.01	0.31	0.73	1.11
Sleep quality (ref very good/good)						
Average	0.73	0.46	-0.50	0.62	0.21	2.51
Not good/very poor	0.63	0.41	-0.71	0.48	0.18	2.25
Smoking (ref non-smoker)	0.88	0.63	-0.17	0.86	0.22	3.58
More than 14 weekly alcoholic drinks (ref =< 14)	4.49	3.66	1.84	0.07	0.91	22.18
Number of mental health care behaviours	1.34	0.27	1.45	0.15	0.90	2.01
Slightly/very overweight (ref under/normal weight)	1.45	0.67	0.81	0.42	0.59	3.59

Note. Data were weighted to the proportions of gender, age, ethnicity, country, and education obtained from the Office for National Statistics. Model 1 included only health behaviours (in the same model), Model 2 additionally adjusted for COVID-19 infection variables, Model 3 additionally adjusted for socio-demographic characteristics, and Model 4 additionally adjusted for pre-existing conditions.

Table S16. Sensitivity analysis: logistic regressions predicting the development of difficulty with cognition from health behaviours, including overweight/obesity status (N = 234) weighted

	Difficulty with cognition					
	OR	SE	T	P	95% CI	95% CI
**Model 1**						
Exercise frequency (ref no exercise)						
<30 min to 2 hours	3.01	2.13	1.55	0.12	0.75	12.11
3+ hours	**6.56**	**5.31**	**2.32**	**0.02**	**1.34**	**32.13**
Days of fresh air (> 15 minutes)	0.92	0.10	-0.78	0.44	0.75	1.13
Sleep quality (ref very good/good)						
Average	1.12	0.61	0.20	0.84	0.38	3.28
Not good/very poor	**3.60**	**2.20**	**2.09**	**0.04**	**1.08**	**11.94**
Smoking (ref non-smoker)	2.88	2.63	1.16	0.25	0.48	17.19
More than 14 weekly alcoholic drinks (ref =< 14)	0.96	0.60	-0.07	0.94	0.28	3.30
Number of mental health care behaviours	**1.57**	**0.31**	**2.27**	**0.02**	**1.06**	**2.32**
Slightly/very overweight (ref under/normal weight)	2.00	1.04	1.34	0.18	0.73	5.53
**Model 2**						
Exercise frequency (ref no exercise)						
<30 min to 2 hours	1.98	1.44	0.94	0.35	0.48	8.22
3+ hours	3.61	3.22	1.44	0.15	0.63	20.73
Days of fresh air (> 15 minutes)	0.91	0.09	-1.01	0.31	0.76	1.09
Sleep quality (ref very good/good)						
Average	1.15	0.62	0.26	0.80	0.40	3.31
Not good/very poor	**3.67**	**2.25**	**2.12**	**0.03**	**1.10**	**12.20**
Smoking (ref non-smoker)	3.14	3.42	1.05	0.29	0.37	26.63
More than 14 weekly alcoholic drinks (ref =< 14)	1.19	0.77	0.27	0.79	0.34	4.21
Number of mental health care behaviours	**1.57**	**0.31**	**2.29**	**0.02**	**1.07**	**2.32**
Slightly/very overweight (ref under/normal weight)	1.30	0.66	0.52	0.60	0.48	3.51
**Model 3**						
Exercise frequency (ref no exercise)						
<30 min to 2 hours	2.47	2.08	1.07	0.28	0.47	12.91
3+ hours	**6.78**	**6.63**	**1.96**	**0.05**	**1.00**	**46.19**
Days of fresh air (> 15 minutes)	0.88	0.09	-1.24	0.21	0.72	1.08
Sleep quality (ref very good/good)						
Average	1.31	0.79	0.44	0.66	0.40	4.28
Not good/very poor	**4.69**	**2.94**	**2.46**	**0.01**	**1.37**	**16.04**
Smoking (ref non-smoker)	2.42	2.36	0.91	0.36	0.36	16.35
More than 14 weekly alcoholic drinks (ref =< 14)	0.87	0.60	-0.20	0.84	0.22	3.39
Number of mental health care behaviours	**2.27**	**0.57**	**3.26**	**<0.001**	**1.39**	**3.71**
Slightly/very overweight (ref under/normal weight)	0.82	0.43	-0.38	0.71	0.29	2.32
**Model 4**						
Exercise frequency (ref no exercise)						
<30 min to 2 hours	2.26	1.93	0.95	0.34	0.42	12.06
3+ hours	6.47	6.37	1.90	0.06	0.94	44.57
Days of fresh air (> 15 minutes)	0.87	0.09	-1.36	0.17	0.71	1.07
Sleep quality (ref very good/good)						
Average	1.22	0.75	0.33	0.74	0.37	4.07
Not good/very poor	**4.83**	**3.12**	**2.44**	**0.01**	**1.36**	**17.16**
Smoking (ref non-smoker)	2.32	2.08	0.94	0.35	0.40	13.44
More than 14 weekly alcoholic drinks (ref =< 14)	0.79	0.57	-0.32	0.75	0.19	3.29
Number of mental health care behaviours	**2.49**	**0.81**	**2.80**	**0.01**	**1.32**	**4.71**
Slightly/very overweight (ref under/normal weight)	0.78	0.43	-0.45	0.65	0.27	2.28

Note. Data were weighted to the proportions of gender, age, ethnicity, country, and education obtained from the Office for National Statistics. Model 1 included only health behaviours (in the same model), Model 2 additionally adjusted for COVID-19 infection variables, Model 3 additionally adjusted for socio-demographic characteristics, and Model 4 additionally adjusted for pre-existing conditions.

Table S17. Sensitivity analysis: logistic regressions predicting the development of difficulty with self-care from health behaviours, including overweight/obesity status (N = 225) weighted

	Difficulty with self-care					
	OR	SE	T	P	95% CI	95% CI
**Model 1**						
Exercise frequency (ref no exercise)						
<30 min to 2 hours	0.54	0.39	-0.85	0.39	0.13	2.23
3+ hours	0.29	0.28	-1.27	0.20	0.04	1.95
Days of fresh air (> 15 minutes)	0.86	0.09	-1.34	0.18	0.70	1.07
Sleep quality (ref very good/good)						
Average	0.72	0.53	-0.45	0.65	0.17	3.01
Not good/very poor	2.41	1.81	1.17	0.24	0.55	10.49
Smoking (ref non-smoker)	0.91	0.60	-0.13	0.89	0.25	3.34
More than 14 weekly alcoholic drinks (ref =< 14)	1.44	0.94	0.56	0.57	0.40	5.20
Number of mental health care behaviours	1.03	0.23	0.11	0.91	0.66	1.59
Slightly/very overweight (ref under/normal weight)	0.72	0.46	-0.51	0.61	0.20	2.55
**Model 2**						
Exercise frequency (ref no exercise)						
<30 min to 2 hours	0.34	0.29	-1.28	0.20	0.06	1.80
3+ hours	0.20	0.21	-1.49	0.14	0.02	1.68
Days of fresh air (> 15 minutes)	0.88	0.11	-1.04	0.30	0.70	1.12
Sleep quality (ref very good/good)						
Average	0.92	0.86	-0.08	0.93	0.15	5.77
Not good/very poor	2.64	2.45	1.04	0.30	0.43	16.31
Smoking (ref non-smoker)	1.27	1.02	0.30	0.76	0.26	6.13
More than 14 weekly alcoholic drinks (ref =< 14)	1.77	1.24	0.81	0.42	0.45	6.99
Number of mental health care behaviours	0.94	0.21	-0.30	0.76	0.61	1.44
Slightly/very overweight (ref under/normal weight)	0.47	0.32	-1.09	0.28	0.12	1.81
**Model 3**						
Exercise frequency (ref no exercise)						
<30 min to 2 hours	0.56	0.50	-0.65	0.52	0.10	3.18
3+ hours	0.17	0.19	-1.59	0.11	0.02	1.51
Days of fresh air (> 15 minutes)	0.89	0.11	-0.93	0.35	0.71	1.13
Sleep quality (ref very good/good)						
Average	0.69	0.66	-0.39	0.69	0.11	4.45
Not good/very poor	1.03	0.94	0.03	0.97	0.17	6.17
Smoking (ref non-smoker)	1.98	1.67	0.81	0.42	0.38	10.31
More than 14 weekly alcoholic drinks (ref =< 14)	2.87	2.23	1.36	0.17	0.63	13.14
Number of mental health care behaviours	0.78	0.24	-0.81	0.42	0.44	1.41
Slightly/very overweight (ref under/normal weight)	0.55	0.40	-0.83	0.41	0.13	2.29
**Model 4**						
Exercise frequency (ref no exercise)						
<30 min to 2 hours	0.67	0.55	-0.49	0.62	0.13	3.33
3+ hours	0.07	0.09	-1.96	0.05	0.00	1.00
Days of fresh air (> 15 minutes)	0.89	0.11	-0.91	0.36	0.70	1.14
Sleep quality (ref very good/good)						
Average	0.39	0.43	-0.86	0.39	0.05	3.34
Not good/very poor	0.89	0.90	-0.12	0.91	0.12	6.48
Smoking (ref non-smoker)	2.03	2.36	0.61	0.54	0.21	19.85
More than 14 weekly alcoholic drinks (ref =< 14)	1.40	1.14	0.41	0.68	0.28	6.91
Number of mental health care behaviours	0.60	0.26	-1.16	0.25	0.25	1.42
Slightly/very overweight (ref under/normal weight)	0.52	0.39	-0.88	0.38	0.12	2.27

Note. Data were weighted to the proportions of gender, age, ethnicity, country, and education obtained from the Office for National Statistics. Model 1 included only health behaviours (in the same model), Model 2 additionally adjusted for COVID-19 infection variables, Model 3 additionally adjusted for socio-demographic characteristics, and Model 4 additionally adjusted for pre-existing conditions.
